# A direct effect of the hematocrit on blood glucose: Evidence from hypoxia- and erythropoietin-treated mice

**DOI:** 10.1126/sciadv.adt7366

**Published:** 2025-04-16

**Authors:** Thomas Scherer, Matthäus Metz, Marianna Beghini, Martin Bilban, Lisa Gensthaler, Andreea C. Luca, Mairam Kaplanian, Sameer Abu Eid, Oliver Koldyka, Martina T. Hackl, Sabine Dürr, Elisa Rivelles, Stefanie S. Schönecker, Lisa Pöltl, Ayperi Kaya, Rime Chami, Laura Nusko, Claudia Tschare, Kathleen Ablaza, Anna-Lena Höbler, Peter Klimek, Michael Leutner, Masayuki Yamamoto, Norio Suzuki, Kerstin Stemmer, Maximilian Zeyda, Daniel Steinacher, Lukas Nics, Antonia M. S. Müller, Thomas H. Helbich, Richard Moriggl, Alexandra Kautzky-Willer, Ursula Windberger, Gerhard Prager, Clemens Fürnsinn

**Affiliations:** ^1^ Division of Endocrinology & Metabolism, Department of Medicine III, Medical University of Vienna, Vienna, Austria.; ^2^ Department of Laboratory Medicine & Core Facilities, Medical University of Vienna, Vienna, Austria.; ^3^ Division of Visceral Surgery, Department of General Surgery, Medical University of Vienna, Vienna, Austria.; ^4^ Department of Laboratory Medicine, Medical University of Vienna, Vienna, Austria.; ^5^ Section for Science of Complex Systems, CeDAS, Medical University of Vienna, Vienna, Austria.; ^6^ Tohoku Medical Megabank Organization, Tohoku University, Sendai, Miyagi, Japan.; ^7^ New Industry Creation Hatchery Center, Tohoku University, Sendai, Miyagi, Japan.; ^8^ Institute for Diabetes and Obesity, Helmholtz Zentrum München, Neuherberg, Germany; and Department of Molecular Cell Biology, Institute of Theoretical Medicine, Faculty of Medicine, University of Augsburg, Augsburg, Germany.; ^9^ Comprehensive Center for Pediatrics, Department of Pediatrics and Adolescent Medicine, Medical University of Vienna, Vienna, Austria.; ^10^ Hans Popper Laboratory of Molecular Hepatology/Division of Gastroenterology and Hepatology, Department of Medicine III, Medical University of Vienna, Vienna, Austria.; ^11^ Department of Biomedical Imaging and Image-guided Therapy, Medical University of Vienna, Vienna, Austria.; ^12^ Department of Transfusion Medicine and Cell Therapy, Medical University of Vienna, Vienna, Austria.; ^13^ Division of Molecular and Structural Preclinical Imaging, Department of Biomedical Imaging and Image-guided Therapy, Medical University of Vienna, Vienna, Austria.; ^14^ Department of Biosciences & Medical Biology, Paris Lodron University of Salzburg, Salzburg, Austria.; ^15^ Center for Anatomy and Cell Biology, Medical University of Vienna, Vienna, Austria.

## Abstract

Blood glucose is lower in mountain dwellers living under low partial oxygen pressure. We show that obese mice maintained under hypoxia exhibit a delayed but distinct decrease in blood glucose with improved insulin sensitivity, which is independent of changes in body weight. This effect of hypoxia is mediated by erythropoiesis and is a direct result of the rising hematocrit, which could be due to erythrocytes acting as carriers of glucose units in the blood. Glucose lowering by the red cell mass is evidenced by a prompt decrease in glycemia in mice receiving a blood transfusion. Furthermore, life under hypoxia as well as treatment with erythropoietin reduce glycemia also in mice expressing the erythropoietin receptor exclusively in hematopoietic cells, which contrasts with previous assumptions attributing metabolic actions of erythropoietin to direct action on nonhematopoietic tissues. Our results provide a rationale for associations between hematocrit and blood glucose in humans under anti-anemic therapy, polycythemia, smoking, and high-altitude exposure.

## Teaser

The mass of red cells circulating in the blood has a direct effect on blood glucose.

## INTRODUCTION

Compared to lowland dwellers, highlanders show lower blood glucose, better insulin sensitivity, and a reduced prevalence of obesity and diabetes (*1*–*5*). This could result from lower partial oxygen pressure at high altitude, but the influence of oxygen shortage is difficult to dissect from that of genetic background, environment, or lifestyle. Although these uncertainties are absent in laboratory rodents exposed to experimental hypoxia, the extent, temporal pattern, and duration of oxygen deprivation still seems to have pivotal influence on the outcome.

Rodent experiments with permanent exposure to hypoxia or with constant exposure for at least several hours per day hint at a biphasic response. Most studies describe an increase in blood glucose during the initial days. This is likely due to elevated sympathetic activity and circulating stress hormones (*6*–*8*), although the response may be masked under extreme hypoxia by the opposing influence of almost complete suppression of food intake (*9*). With prolonged exposure and progressive adaptation to the hypoxic environment, however, the initial increase in glycemia seems to turn into a decrease (*5*–*7*, *9*–*11*). Diminished body weight surely contributes to this delayed fall of blood glucose, but the larger part of the response obviously relies on a not yet identified mechanism other than weight reduction (*12*–*14*). Although available information thus points at opposing early and delayed responses, uncertainties remain because most pertinent studies have examined blood glucose at unclear stages of this putative biphasic pattern and failed to dissect indirect effects via hypoxia-induced loss of appetite from other mechanisms (*9*, *12*, *15*–*17*). Adding to the lack of clarity, many handheld glucose meter devices routinely used in mice and mountaineers are disturbed not only by oxygen tension and temperature at high altitude but also by a nonnegligible sensitivity to the hematocrit of the blood sample (*18*–*22*).

Bypassing these uncertainties, the present study built on mouse experiments carefully designed for pinpointing the hypoxia-adapted phenotype and its underlying mechanisms in the absence of influence from weight changes and analytical artifacts. We provide straightforward evidence that the elevated hematocrit not only is directly responsible for glucose lowering in mice living under hypoxia but also is generally a substantial modulator of blood glucose, which seems, at least in part, attributable to terminally differentiated erythrocytes acting as glucose carriers in the blood. Our finding has potential to conclusively explain concomitant changes in hematocrit and glycemia as they are observed not only in humans at high altitude (*5*) but also in many clinical circumstances including smoking (shown in this study), anti-anemic therapy (*23*, *24*), testosterone treatment (*25*, *26*), and polycythemia (*27*).

## RESULTS

### Life under hypoxia lowers blood glucose and improves insulin sensitivity

**Fig. 1.**
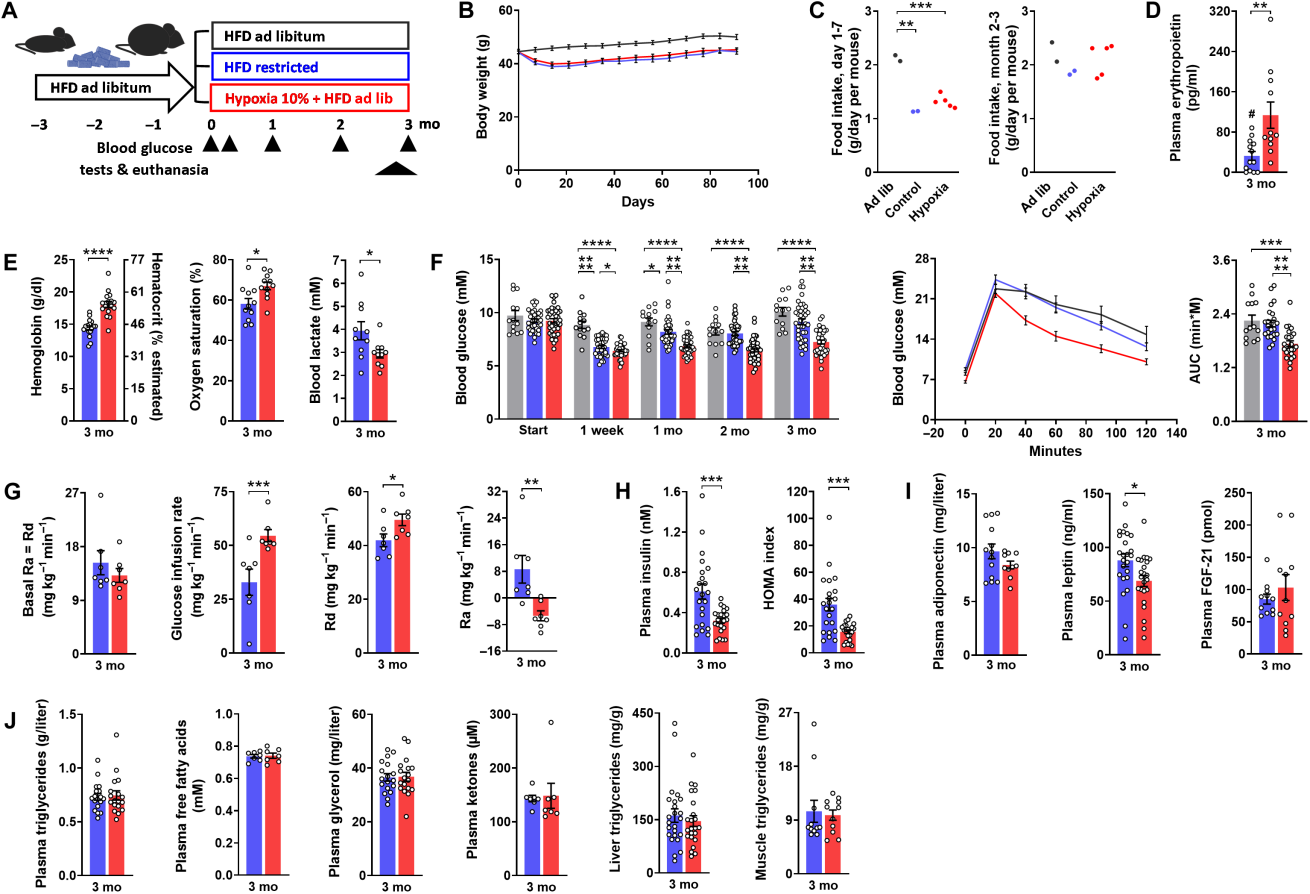
Life under hypoxia lowers blood glucose and improves insulin sensitivity. Metabolic characteristics of male obese mice, which, after 3 months on HFD, were exposed to hypoxia for another 3 months (10% O_2_, red). They were compared to control groups at normal air with free access to food (gray) or with restricted access to food (blue) so to maintain a weight curve mimicking that of the hypoxia-exposed mice. Graphs depict (**A**) experimental protocol; (**B**) weight curves; (**C**) food intake (cage-wise); (**D**) plasma EPO (#, not detectable in 3 of 13 controls); (**E**) hemoglobin/hematocrit, blood oxygen saturation, and blood lactate; (**F**) basal blood glucose and glucose excursion during a glucose tolerance test with the corresponding area under the curve (AUC) [1.5 g/kg intraperitoneally (ip)]; (**G**) basal rate of disappearance [Rd = equal to basal rate of appearance (Ra)], glucose infusion rate, insulin-stimulated Rd, and insulin-stimulated Ra from a euglycemic-hyperinsulinemic clamp test; (**H**) basal plasma insulin and HOMA index; (**I**) plasma adiponectin, leptin, and FGF-21; and (**J**) plasma triglycerides, free fatty acids, glycerol and ketones, and ectopic triglycerides in the muscle and liver. mo, months; ad lib, ad libitum. Means ± SEM; *P < 0.05; **P < 0.01; ***P < 0.001; ****P < 0.0001, Student’s *t* test.

Given the uncertainties afflicting preceding studies, it was imperative to initially pin down the true effect of hypoxia on blood glucose in consideration of exposure time, weight dependence, and hematocrit sensitivity of glucose meters. Male mice, which were obese after 3 months on high-fat diet (HFD), were maintained under 10% O_2_ for another 3 months and compared to restrictedly fed (i.e., weight-matched), as well as to freely feeding controls (Fig. 1A). Note that mice had to be removed from hypoxia and were at normal air for 2 to 4 hours before any sampling of blood or tissues, implicating that our results describe the adaptive response to life under hypoxia but not the acute effect of oxygen shortage.

During the initial days under hypoxia, mice appeared inactive, consumed less food, and lost weight. This response faded within the first month so that hypoxia-exposed mice thereafter did not fall back further in weight versus their ad libitum–fed controls (Fig. 1, B and C).

After 3 months under hypoxia, plasma erythropoietin (EPO) was elevated along with a 10% rise in the hematocrit, an increase as seen in humans after a prolonged stay at 3.500 to 4.000 m of altitude (*28*). A higher oxygen transport capacity thus obviously accounted for increased oxygen saturation and decreased blood lactate in hypoxia-adapted mice (Fig. 1, D and E). Whereas a drop in blood glucose seen after 1 week under hypoxia was largely attributable to weight loss, more prolonged maintenance under hypoxia unmasked marked weight-independent improvements of glycemia and glucose tolerance (Fig. 1F), which persisted 24 hours after removal from the hypoxic environment (fig. S1A). Although the basal rate of glucose disappearance (= rate of appearance) was unchanged, euglycemic-hyperinsulinemic clamp tests revealed improvements in peripheral and hepatic insulin sensitivity, as also reflected by reduced plasma insulin and a lower HOMA (Homeostasis Model Assessment, a readout for insulin sensitivity based on basal glycemia and insulinemia; Fig. 1, G and H, and fig. S1B). With regard to circulating modulators of insulin sensitivity, the insulin-sensitizing peptide adiponectin showed a hypoxia-induced trend toward lower plasma concentrations (*P* = 0.14), as would fit with the inverse association with the hematocrit described in humans (*29*, *30*). Plasma fibroblast growth factor-21 (FGF-21) was unaffected and slightly lower plasma leptin in hypoxia-treated mice was the correlate of a modest reduction in relative fat mass, as suggested by intraindividual associations (Fig. 1I and fig. S1, C and D). Neither leptin nor % fat mass were intraindividually associated with glycemia or glucose tolerance (*r* < 0.34 each in hypoxia and control group), which excluded relevance of the subtle changes in body composition to glucose lowering. Neither circulating lipids, nor tissue triglycerides, or liver weight were affected by hypoxia (Fig. 1J and fig. S1E).

### EPO treatment mimics the glucose lowering effect of hypoxia

**Fig. 2.**
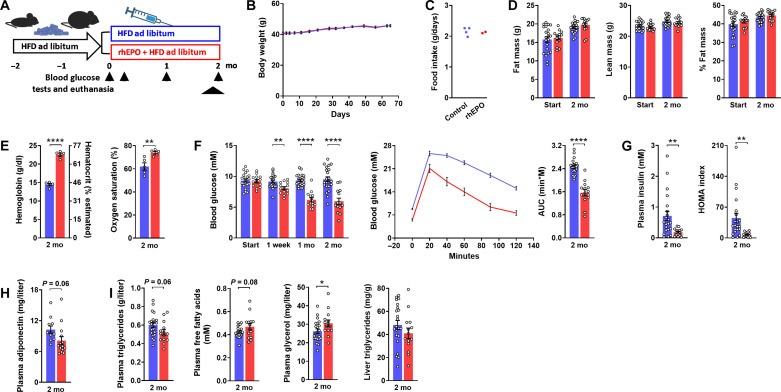
EPO treatment mimics the glucose lowering effect of hypoxia. Metabolic characteristics of male obese mice, which, after 2 months on HFD, were treated for another 2 months with three intraperitoneal doses per week of epoetin theta (rhEPO; 300 U/kg; red) or the vehicle (blue). Graphs depict (**A**) experimental protocol; (**B**) weight curves; (**C**) food intake (cage-wise); (**D**) body composition; (**E**) hemoglobin/hematocrit and blood oxygen saturation; (**F**) basal blood glucose and glucose excursion during a glucose tolerance test with the corresponding AUC (1.5 g/kg ip); (**G**) basal plasma insulin and HOMA index; (**H**) plasma adiponectin; and (**I**) plasma triglycerides, free fatty acids, glycerol, and ectopic triglycerides in the liver. Means ± SEM; *P < 0.05; **P < 0.01; ****P < 0.0001, Student’s *t* test.

Amelioration of hyperglycemia by regular injections of recombinant human EPO (rhEPO) has been described in rodents (*31*–*33*), but similar to previous studies about hypoxia conclusions were hampered by an unaccounted influence of weight differences and/or hematocrit-sensitive glucose meters. For initial clarification of the true influence of rhEPO, male obese mice after 2 months on HFD received three weekly intraperitoneal doses of rhEPO (300 U/kg) for another 2 months (Fig. 2A). rhEPO failed to affect body weight, food intake, and body composition (Fig. 2, B to D) so that splitting of the controls into restrictedly and ad libitum–fed groups was not required. This resembled previous results from rats (*32*) but was at variance to impaired weight gain under similar or higher dosage in mouse studies (*31*, *33*, *34*). The response to rhEPO strongly resembled that to hypoxia with significant effects on hematocrit, blood oxygen saturation, blood glucose, glucose tolerance, plasma insulin, and HOMA, as well as with a nonsignificant trend for plasma adiponectin (Fig. 2, E to H). Minor changes in circulating lipids hinted at subtle stimulation of lipolysis (Fig. 2I). A single dose of rhEPO failed to acutely affect blood glucose and glucose lowering was absent in insulin-deficient mice treated with rhEPO, which was in line with an insulin-dependent mode of action (fig. S2). Effects of rhEPO on glucose homeostasis as seen in male obese mice were basically the same in lean males (fig. S3) and in obese females (fig. S4).

In summary, the metabolic phenotype arising from a prolonged stay under hypoxia was thus mimicked by regular injections of rhEPO, which strongly pointed at endogenous EPO as a crucial mediator.

### Glucose lowering does not require EPO action in nonhematopoietic tissues

Aiming to develop hypotheses about events that mediate hypoxia-induced glucose lowering downstream of EPO, we performed a whole transcriptome analysis on tissues from obese hypoxia-exposed mice and their weight-matched controls. Gene set enrichment analysis (GSEA) revealed distinct up-regulation by hypoxia of gene sets typically addressed by EPO [i.e., JAK-STAT (Janus kinase–signal transducer and activator of transcription) pathway and hematopoietic cell lineage] in skeletal muscle, which was less pronounced in liver and absent in fat. Furthermore, gene sets involved in oxidative phosphorylation and fatty acid metabolism were distinctly up-regulated in muscle with an opposing effect in the liver and no effect in fat (fig. S5). The pattern in skeletal muscle appeared to fit with evidence for EPO being a stimulator of mitochondrial biogenesis and fat oxidation (*35*–*38*), which, in turn, has been associated with amelioration of lipotoxicity and insulin sensitization (*39*, *40*). In our mice, however, hypoxia did not affect mitochondrial density, oxidative fuel selection ex vivo, or the expression of mitochondrial biogenesis genes (except for a minor increase in the muscle expression of *Ppargc1a*; fig. S6), which was in line with a recent report of unchanged substrate fluxes into the tricarboxylic acid cycle (*9*). Another putative direct target organ of EPO is the brain, where EPO seems to interfere with leptin signaling (*41*–*43*), but a central nervous system infusion of rhEPO into the third ventricle also remained without effects on blood glucose (fig. S7).

EPO’s effects on fuel metabolism, including in particular those on mitochondrial biogenesis, are currently attributed to direct actions on nonhematopoietic tissues via a receptor heterodimer composed of the EPO receptor (EpoR) and the β-common receptor (βCR) (*35*, *43*). However, ARA290 (cibinetide), which specifically activates the heterodimeric receptor but not the homodimeric EpoR mediating hematopoiesis, lacked any effect in obese mice, arguing against a role of EpoR/βCR in glucose lowering by hypoxia and EPO in our mice (fig. S8).

**Fig. 3.**
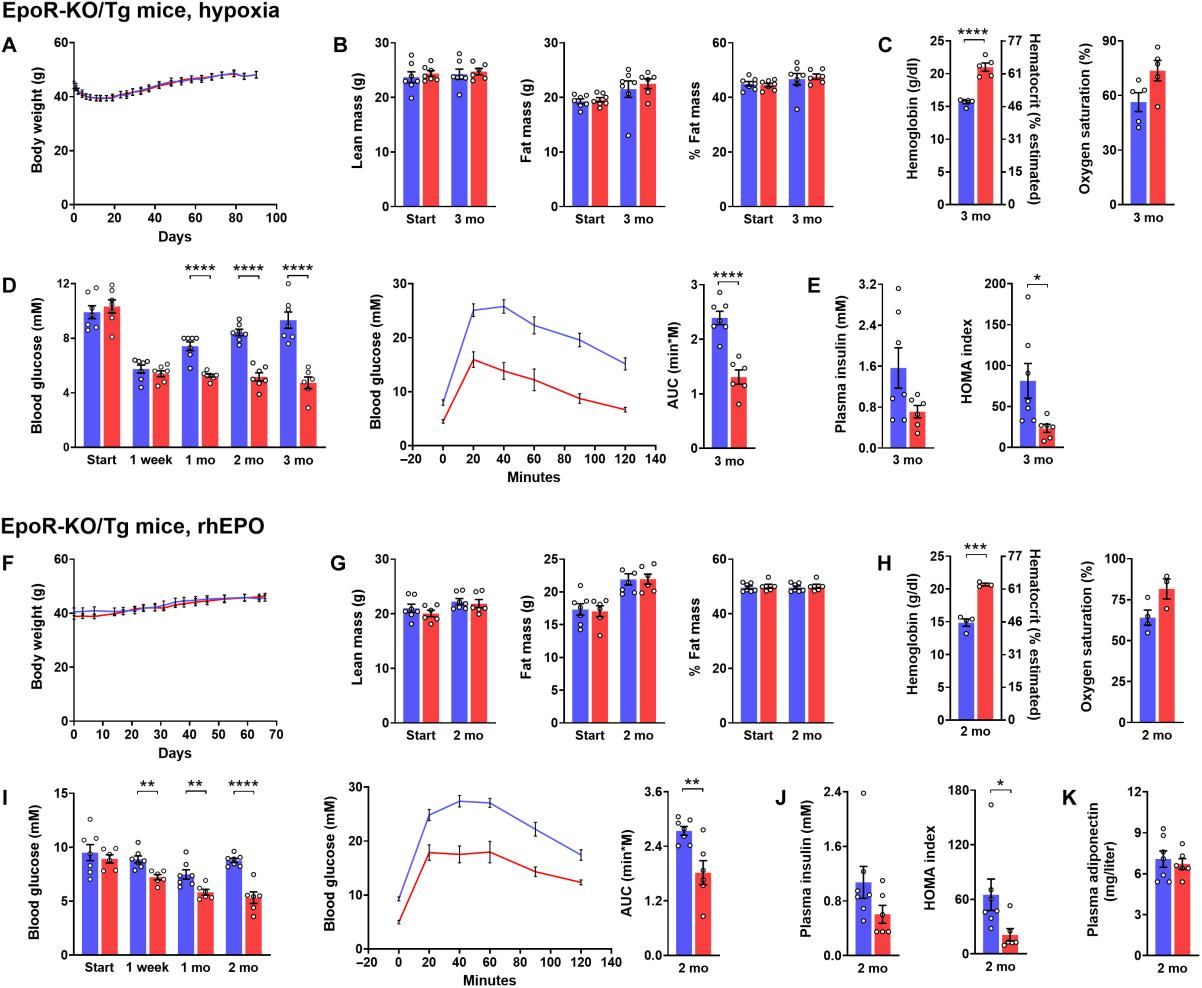
Glucose lowering does not require EPO signaling in nonhematopoietic tissues. Metabolic characteristics of male obese EpoR-KO/Tg mice, which, after 3 months of high-fat feeding, were exposed to hypoxia (10% O_2_) for another 3 months (**A** to **E**) or, after 2 months of high-fat feeding, were treated with three intraperitoneal doses per week of epoetin theta (rhEPO; 300 U/kg) for another 2 months (**F** to **K**). Treated groups are shown in red. Corresponding controls were maintained at normal air or treated with the vehicle (blue), and were fed restrictedly so to maintain a weight curve mimicking that of their respective treated counterparts. Graphs depict [(A) and (F)] weight curves, [(B) and (G)] body composition, [(C) and (H)] hemoglobin/hematocrit and blood oxygen saturation, [(D) and (I)] basal blood glucose and glucose excursion during a glucose tolerance test with the corresponding AUC (1.5 g/kg ip), [(E) and (J)] basal plasma insulin and HOMA index, and (K) plasma adiponectin (for rhEPO-treated mice). Means ± SEM; *P < 0.05; **P < 0.01; ***P < 0.001; ****P < 0.0001, Student’s *t* test.

To lastly clarify whether direct EPO action on nonhematopoietic tissues has any role in glucose lowering, we exposed HFD-obese male EpoR-knockout (KO)/transgenic (Tg) mice, which express EpoR only in the hematopoietic lineage (*44*), to hypoxia and EPO treatment protocols previously applied to wild-type (WT) mice (see Figs. 1 and 2). Similar to WT mice, EpoR-KO/Tg mice showed an initial decrease in body weight in response to hypoxia but not rhEPO (Fig. 3, A and F). Whereas weight-neutral changes in body composition and plasma adiponectin were not observed (Fig. 3, B, G, and K), the hematocrit of EpoR-KO/Tg mice increased (as expected due to the presence of EpoR in their bone marrow and spleen; Fig. 3, C and H). Lack of EpoR in nonhematopoietic tissues did in no way impair the efficacy of hypoxia and rhEPO to ameliorate hyperglycemia, glucose intolerance, and insulin resistance, clearly implicating that direct EPO action on nonhematopoietic tissues was not required for glucose lowering (Fig. 3, D, E, I, and J).

### A direct impact of the hematocrit on blood glucose

**Fig. 4.**
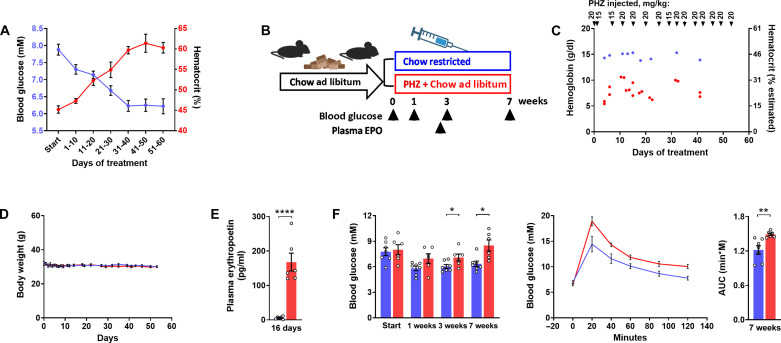
Hematocrit-induced glucose lowering: Time dependence and anemic mice. (**A**) Time-dependent changes in blood glucose (blue) and hematocrit (red) of male obese mice, which, after 2 months of high-fat feeding, were treated with three intraperitoneal doses per week of epoetin theta (300 U/kg). Blood glucose values are the average of three measurements within the indicated 10-day time interval. Hematocrit was measured once per 10 days. (**B** to **F**) Metabolic characteristics of lean male mice fed carbohydrate-rich diet (Chow) and receiving repeated injections of PHZ so to induce anemia with a hematocrit around 25% (red). Controls were injected with the vehicle and were fed restrictedly so to maintain a weight curve mimicking that of the PHZ-treated mice (blue). Graphs depict (B) experimental protocol, (C) hemoglobin/hematocrit and dosing of PHZ, (D) weight curves, (E) plasma EPO, and (F) basal blood glucose and glucose excursion during a glucose tolerance test with the corresponding AUC (1.5 g/kg ip). Means ± SEM; *P < 0.05; **P < 0.01; ****P < 0.0001, Student’s *t* test.

The findings from EpoR-KO/Tg mice raised the question whether the mass of circulating red cells itself affects glycemia. When we juxtaposed the time-dependent rise of the hematocrit versus the fall in glycemia in rhEPO-treated obese mice, we found a perfect match with an approximately linear increase/decrease during the initial 40 days followed by a plateau thereafter (Fig. 4A). The possibility that the hematocrit drives changes in glycemia was further supported by mice treated with phenylhydrazine (PHZ), which induces hemolysis and anemia while triggering a counterregulatory rise in circulating EPO (*45*, *46*). The hematocrit of PHZ-treated mice was maintained at roughly 25% for 7 weeks, whereas vehicle-injected controls were fed restrictedly to avoid any difference in body weight (Fig. 4, B to D). Despite a 28-fold increase in plasma EPO, anemic mice showed elevated glycemia and glucose intolerance, which ruled out glucose lowering action of EPO while corroborating a strong inverse association of blood glucose with the concurrent hematocrit (Fig. 4, E and F).

**Fig. 5.**
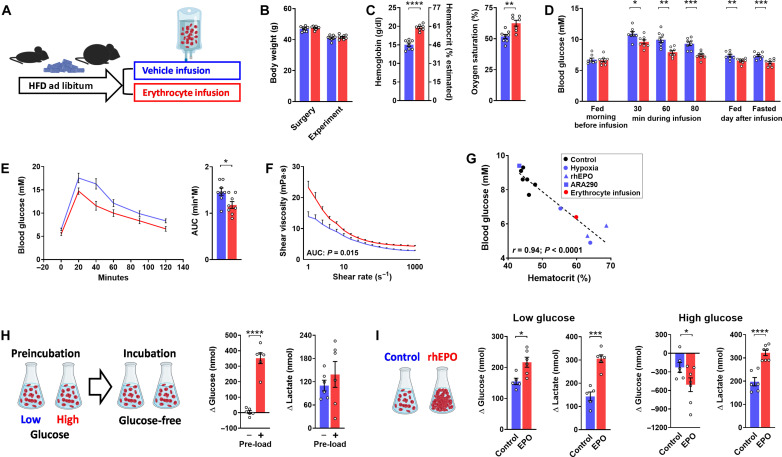
Erythrocyte infusion and glucose handling by isolated erythrocytes. (**A** to **F**) Metabolic characteristics of male obese mice, which, after 3 months of high-fat feeding (HFD), were infused with erythrocytes from donor mice (red). Controls were infused with the vehicle (blue). Graphs depict (A) experimental protocol; (B) body weight at surgery and at day of the experiment; (C) hemoglobin/hematocrit and blood oxygen saturation 4 hours after the infusion; (D) basal blood glucose on the morning before, during, and after the 80-min infusion period, as well as on the next day in the fed and 4-hour fasted state; (E) glucose tolerance test with the corresponding AUC (1.5 g/kg ip) performed 2 hours after the infusion; and (F) shear viscosity of blood 24 hours after the infusion. (**G**) Association between the means of basal blood glucose and hematocrit in all groups of male HFD-obese mice from the present study, including both WT and EpoR-KO/Tg mice. The graph includes data from mice treated with hypoxia (blue circles), epoetin theta (rhEPO; blue triangles), ARA290 (blue quadruple), or acutely infused with donor erythrocytes (red circle). Black circles are data from the various control groups; *r* and *P* inside the graph by Pearson’s correlation analysis. (**H**) Net release of glucose and lactate from isolated mouse erythrocytes exposed to glucose-free incubation medium after preincubation for 1 hour with high glucose (16.5 mM, red; “pre-load”) or low glucose (0.55 mM, blue). (**I**) Net release of glucose and lactate from erythrocytes isolated from 200 μl of blood from mice treated with the vehicle (blue) or with three weekly intraperitoneal doses of epoetin theta (rhEPO; 300 U/kg; red). Erythrocytes were incubated with low or high glucose immediately after collection (1.1 mM, left; 22 mM, right). Means ± SEM; *P < 0.05; **P < 0.01; ***P < 0.001; ****P < 0.0001, Student’s *t* test.

We lastly pinned down the role of the hematocrit by venous infusion of obese mice with donor erythrocytes, which raised the hematocrit to 60% as compared to 46% in weight-matched, vehicle-infused controls (Fig. 5, A to C). The acute rise in the circulating red cell mass significantly lowered blood glucose in the erythrocyte-infused mice already after the initial 30 min of the 80-min infusion period, when less than half of the erythrocytes had been administered. This response became more pronounced during the further course of the infusion. Two hours after the infusion, glucose tolerance was improved, and fasted as well as fed glycemia were still lower in the erythrocyte-infused than in the control mice on the next day (Fig. 5, D and E). Erythrocyte infusion elevated blood viscosity (Fig. 5F), which is believed to slow blood flow and hence may increase retention time and glucose extraction in capillaries. However, a parallel experiment with infusion of the high–molecular weight colloid alginate [sodium alginate (15 g/liter) in saline] known to increase the viscosity of blood (*47*) did not provide evidence for a viscosity-dependent reduction of glycemia (fig. S9).

Juxtaposition of the means of hematocrit versus basal blood glucose from all experiments performed with male HFD-obese mice further confirmed a close association of blood glucose with the concurrent hematocrit, irrespective of whether the hematocrit was modulated by hypoxia, rhEPO, ARA290, or acute erythrocyte infusion (Fig. 5G).

### Erythrocytes as carriers of glucose units

Capable of glycogen storage, erythrocytes have been proposed to buffer fluctuations in blood glucose and to act as glucose carriers between organs (*48*–*51*). We preincubated aliquots of erythrocytes collected from male obese mice with low or high glucose for 1 hour (0.55 and 16.5 mM, respectively). After replacing the medium with glucose-free phosphate-buffered saline (PBS), only erythrocytes “preloaded” with high glucose showed net release of an amount of glucose as would cause a rise in glycemia by ~2 mM (Fig. 5H), which confirmed that erythrocytes can store and release relevant quantities of glucose.

We then collected erythrocytes from 200-μl aliquots of blood from obese mice treated with rhEPO, which, in comparison to vehicle-treated controls, were more in mass but exposed to much lower glycemia at the time of collection (for rhEPO dosage and in vivo effects see in Fig. 2). Exposed to low glucose immediately after collection (1.1 mM), the erythrocytes from rhEPO-treated mice released significantly more glucose and lactate than those from control mice (Fig. 5I), in sum equivalent to a difference in energetic value of 12.4 ± 0.4 versus 7.7 ± 0.5 μmol of adenosine triphosphate (*P* < 0.0001). Vice versa, net glucose uptake was elevated in erythrocytes from rhEPO-treated versus control mice, when incubated after collection with high glucose (22 mM; Fig. 5I). After subtraction of the respective amounts of glucose converted to lactate, this related to a 2.7-fold increase in glucose units that entered pathways other than glycolysis, as may predominantly have been glycogen storage (*50*, *51*). Note that the amount of lactate released under all conditions reflected the erythrocyte mass, suggesting constant energy requirements of the red cells. Together, these data substantiate previous findings that erythrocytes take up and release amounts of glucose that relevantly influence circulating blood glucose.

### Increased hematocrit and reduced blood glucose in young smokers

**Fig. 6.**
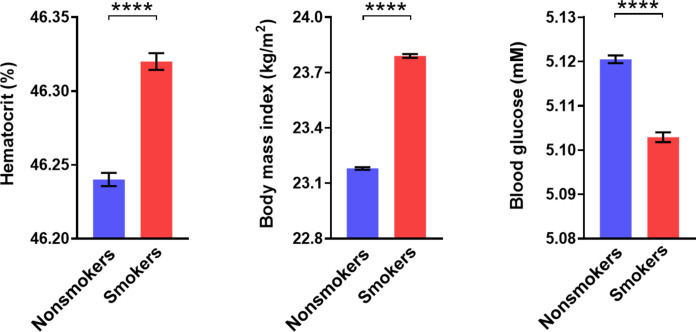
Hematocrit and blood glucose in nonsmokers and smokers. Hematocrit, BMI, and blood glucose in male adolescent nonsmokers (blue; *n* = 302,829) versus smokers (red; *n* = 194,605). Note that scaling of the *y* axes does not start at 0. Means ± SEM; ****P < 0.0001, Student’s *t* test.

Experimental long-term interventions as applied to mice are hardly doable in humans, but pertinent changes in glycemia and insulin sensitivity have been described in association with hematocrit changes caused by altitude, Chuvash polycythemia, and therapeutic administration of rhEPO or testosterone (*5*, *23*–*25*, *27*). Because smoking can be regarded as another “intervention” that raises hematocrit and hemoglobin (*52*, *53*), we compared smokers versus nonsmokers examined in the course of compulsory military conscription in Austria (*n* = 497,434). This population seemed particularly suited for our purpose because young age (>97% of subjects 18 years old) implicated a short history of smoking and hence little influence from long-term sequelae. Our analysis revealed that not only the hematocrit but also the body mass index (BMI) was higher in smokers than nonsmokers. The smoking-induced increase in the hematocrit was accompanied by significantly lower glycemia, which even exceeded the well-known opposing influence of the BMI (Fig. 6).

## DISCUSSION

Many studies have investigated the changes in blood glucose of rodents exposed to experimental hypoxia, but diversity of protocols, use of hematocrit-sensitive glucose meters, and an unclear contribution of indirect action via changes in appetite and body weight have so far hampered straightforward conclusions. Carefully applying a protocol that avoids any influence of body weight differences and hematocrit-sensitive devices (*12*), our results confirmed a delayed but clear-cut decrease in blood glucose in mice adapting to life under hypoxia. This response was mimicked by regular treatment with rhEPO and went along with insulin sensitization, but the metabolic phenotype of the treated mice deviated from what is usually observed after insulin sensitization by other interventions, with untypical attributes including the induction of hypoglycemia in lean mice and the lack of accompanying effects on circulating lipids or ectopic triglycerides in the obese.

In regard to the mechanism, via which hypoxia decreased blood glucose, our results pointed at EPO-mediated erythropoiesis as an essential event and identified the prevailing hematocrit as a previously unknown and immediate modulator of blood glucose. Establishing the sheer amount of red cell mass as the crucial mediator of hypoxia- and EPO-induced glucose lowering could mark a paradigm shift because the effects of EPO on fuel metabolism were hitherto primarily explained by direct effects on nonhematopoietic tissues like fat, liver, brain, pancreatic β cells, and muscle (*34*–*36*, *43*, *54*–*58*). Even if not responsible for reduced blood glucose in our protocols, importance of EpoR in these tissues for the homeostasis of body weight and blood glucose has been demonstrated in studies using EpoR-KO/Tg mice (*34*), as well as in mice with EpoR deleted specifically in adipocytes (*55*), neural cells (*59*), or pancreatic β cells (*56*). These effects via nonhematopoietic tissues, however, are generally attributed to EpoR/βCR heterodimers, whereas hematopoiesis is mediated by homodimers of EpoR. Dependence on different receptor dimers could explain why we found the hematocrit-induced reduction of blood glucose at a lower dosage of rhEPO and after longer treatment periods than usually associated with hematocrit-independent actions.

Regarding downstream events that possibly connect high hematocrit with low glycemia, our findings corroborate previous evidence that glucose units are transported not only in the circulation as blood glucose but also in erythrocytes, which show distinct changes in glycogen content dependent on the ambient glucose concentration and after a single passage through an organ (*50*, *51*, *60*). Clinical importance of carbohydrate delivery by erythrocytes has been demonstrated with regard to brain function of humans with glucose transporter-1 (GLUT1) deficiency, which included evidence for direct substrate transfer from the red blood cell to the endothelium (*48*). Our in vitro incubation experiments further showed that a larger mass of red blood cells converts more glucose into lactate, implicating that an elevated hematocrit means potential for transport and delivery of more glucose units in the form of not only erythrocyte glycogen but also as lactate and, as indicated by others (*48*), as pyruvate. Transport of glucose units in molecular entities other than free glucose should thus, at least in part, explain the observed reduction in blood glucose. Although we did not directly quantitate the contribution to peripheral carbohydrate delivery, it is of note that basal glucose disposal of hypoxia-adapted mice was not diminished despite lower glycemia, which is compatible with the possibility of increased supply of glucose units that do not appear in blood glucose. Unimpaired supply under reduced glycemia would also fit with our observations that elevated hematocrit hardly affected lipid parameters of obese mice and improved glucose tolerance also in lean mice. Furthermore, “hidden” glucose units in the blood could, at least in part, account for the increase in insulin-stimulated glucose disposal under a clamped blood glucose concentration, which, in turn, would explain the different phenotypes arising from hypoxia versus other interventions that trigger insulin sensitization in the euglycemic clamp.

**Fig. 7.**
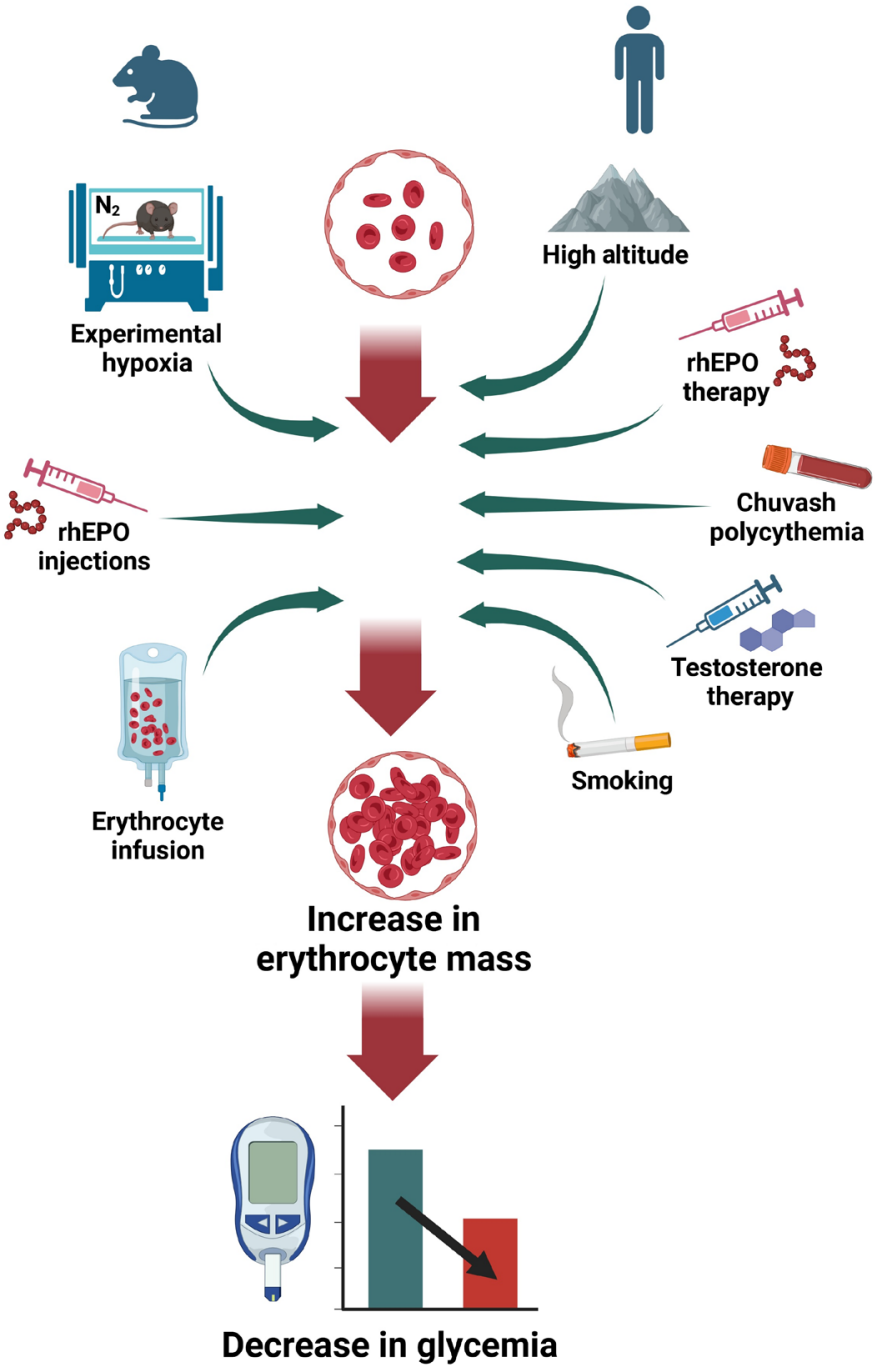
Evidence that a high hematocrit lowers blood glucose in mice and humans. Graphical summary of evidence indicating that a high hematocrit lowers blood glucose. (**Left**) Experimental interventions in rodents as applied in the present study, which caused an increase in the hematocrit accompanied by a decrease in blood glucose: maintenance under hypoxia, treatment with rhEPO, and infusion of donor erythrocytes. (**Right**) Conditions under which an increase in the hematocrit is associated with reduced glycemia in humans: a stay at high altitude, anti-anemic therapy with rhEPO, Chuvash polycythemia, testosterone therapy, and smoking.

Albeit currently available data do not allow comparison of the magnitude of effects, there is substantial evidence to suggest that hematocrit-dependent glucose lowering found in mice could also be relevant to humans. A trend toward loss of insulin sensitivity was found in five subjects after acute reduction of the hematocrit by erythro-apheresis (*61*), and several conditions that increase the hematocrit simultaneously reduce blood glucose in humans (graphically summarized in Fig. 7). Apart from decreased blood glucose in mountain dwellers (*5*), we showed in adolescent males that the elevated hematocrit typically found in smokers (*52*, *62*) went along with reduced glycemia, which even excelled the established opposing influence of the BMI. Furthermore, others have reported that anti-anemic treatment with rhEPO reduces glycemia, improves insulin sensitivity, and raises the frequency of hypoglycemic events (*23*, *24*, *63*–*65*). Likewise, testosterone therapy and Chuvash polycythemia are characterized by a rise in the hematocrit along with reduced blood glucose and HbA1c (*25*–*27*, *66*, *67*). It even appears possible that the elevated hematocrit induced by sodium-glucose cotransporter-2 (SGLT2) inhibitors could contribute to their antidiabetic action, albeit their predominant mode of action undoubtedly remains glucose excretion via the urine (*68*, *69*).

In conclusion, we have found in mice a previously unknown, direct, and immediate influence of the hematocrit on blood glucose, which are both among the most frequently measured blood parameters in clinical medicine. By clarifying the crucial role of the hematocrit, we uncovered the proximal steps of the mechanism, which connects a prolonged stay under hypoxia to the lowering of blood glucose. Our results help explain a multiplicity of circumstances, under which an increased mass of circulating erythrocytes is associated with reduced glycemia and with a lower prevalence of diabetes mellitus in humans.

## MATERIALS AND METHODS

### Experimental design

The study was of exploratory design. The aims were to pinpoint the metabolic phenotype of mice living under hypoxia and to stepwisely develop mechanistic hypotheses about how living under hypoxia affects blood glucose.

### Mouse models

Mice were housed in groups of ~5 per cage at room temperature under an artificial 12-hour dark/12-hour light cycle and had free access to tap water. Husbandry and manipulations were in accordance with good practice and institutional and national guidelines.

Male and female WT C57BL/6JRj mice from the breeding facilities of the Medical University of Vienna (Himberg, Austria) were delivered at an age of ~8 weeks. Transgene-rescued EpoR-null mutant mice [EpoR-KO/Tg mice; B6.Cg-Epor<tm1Liz> Tg(Gata1-Epor)AMym/MymRbrc] expressing EpoR exclusively in the hematopoietic lineage have been established earlier (*44*). They were bred in-house starting off with embryos obtained from RIKEN Bio Resources Center (RBRC00985; Kyoto, Japan). Their genotype was confirmed by polymerase chain reaction (PCR) of DNA extracted from ear clips using the following primers: (i) 5′-GAGTTCTCTGCGCTCACACAC-3′, (ii) 5′-ACACGTCCACTTCATATCGG-3, (iii) 5′-AAGTATCCATCATGGCTGATG-3′, and (iv) 5′-ATACCAGCTCGAGGGTGAGTC-3′ (primer set 1+2 for EpoR WT; 1+3 for EpoR KO; 2+4 for Tg).

Mice were fed conventional carbohydrate-rich diet (chow diet; Rod16-A, LASvendi, Soest, Germany) or HFD (D12492, 60% calories as fat, Research Diets, New Brunswick, NJ, USA), the latter known to cause obesity and glucose intolerance in the C57BL/6 strain (*12*, *70*). Mice were allocated to their experimental groups by matching, aiming at comparable group means for body weight, basal glycemia, and, if available, body composition (values for matching collected in the last week before starting the experimental intervention). All manipulations and analytical procedures used mice/samples from the different groups in a mixed sequence so to avoid confounders and artifacts.

### Control groups and restricted feeding

In all protocols involving the administration of fluids with dissolved agents, corresponding control mice were treated in the same way with the vehicle. If an experimental intervention affected weight gain, the control group was fed restrictedly with one portion of food per day so to obtain a mean weight curve as similar as possible to the curve of their treated counterparts. To this end, mice were weighed every few days and food portions were adjusted if necessary. In some experiments, an additional control group with free access to food was run for comparison. This technique served to dissect indirect effects mediated by appetite and weight differences from other actions and has been applied at our laboratory earlier (*12*, *71*).

### Exposure to hypoxia

Cages were placed in an A-chamber from BioSpherix (ProOx 110; Parish, NY, USA) equipped with oxygen sensors and controllers, which allowed maintenance of the target oxygen concentration in the atmosphere by controlled inflow of appropriate amounts of nitrogen. During the initial 3 days the oxygen concentration was lowered stepwisely, before the final target concentration of 10% O_2_ was continuously maintained from day 3 onward. The chamber was briefly opened once daily for husbandry purposes. Before any sampling of blood glucose and tissues, the box remained open for 2 to 4 hours and mice were at normoxic animal laboratory air. The results therefore describe the adaptive response to a prolonged stay under hypoxia but not the acute effect of current oxygen shortage. For the study of obese C57BL/6JRj mice, HFD was fed for 3 months before treatment with hypoxia started.

### Treatment with rhEPO or ARA290 (cibinetide)

Brief experiments were performed to examine the short-term effects of a single intraperitoneal injection of rhEPO (epoetin theta from ratiopharm, Ulm, Germany). Most experiments using rhEPO, however, dealt with prolonged regular treatment. To this end, mice received three intraperitoneal injections per week at intervals of at least 48 hours (usually Monday-Wednesday-Friday) of rhEPO diluted with saline or ARA290 (generously provided by Airam Pharmaceuticals, Tarrytown, NY, USA) dissolved in PBS at pH 7.45 (injection volume: 3 μl/g body weight). Injected doses of rhEPO were 300 U/kg, whereas ARA290 was injected at doses of 75 μg/kg during the initial month and 150 μg/kg for the following 2 months. All tests and measurements were 48 hours after the last preceding injection. For the study of obese C57BL/6JRj mice, HFD was fed for 2 months before the treatment with rhEPO or ARA290 started. Lean mice on chow diet were of similar age as the obese at the time of rhEPO treatment.

### Induction of insulin deficiency

For the induction of insulin deficiency, 4-month-old chow-fed male C57BL/6JRj mice were injected with streptozotocin (Sigma-Aldrich, St. Louis, MO, USA) for 2 consecutive days [150 mg/kg intraperitoneally per day). Most individuals developed severe hyperglycemia, and only these were used for the experiments. For inclusion, the average blood glucose in the fed state from three independent measurements on days 8, 9, and 10 after streptozotocin treatment had to be >16.5 mM.

### Treatment with PHZ

The hemolytic drug PHZ (Sigma-Aldrich) was dissolved in saline (5 g/liter) for repeated intraperitoneal injection in 7-month-old chow-fed male C57BL/6JRj mice. At intervals of 2 or 3 days, the hematocrit was measured in arbitrarily selected individuals. Results were used for determination of the subsequent PHZ dose and dosing interval, always aiming at maintenance of the hematocrit at roughly 25% [which was lastly achieved with ~2 injections per week of PHZ (15 or 20 mg/kg)].

### Body composition

A whole body composition analyzer was used for the determination of body composition (lean/fat mass; EchoMRI, Houston, TX, USA). Food was not withdrawn before these measurements.

### Glycemia, insulinemia, HOMA, and glucose tolerance test

For the determination of basal glycemia and insulinemia, mice were fasted in the morning. Four hours later, their tails were pricked with an injection cannula and blood glucose was determined with a hematocrit-insensitive handheld glucose meter (described in detail below in the section “Analysis of blood and plasma samples”). For the determination of basal plasma insulin, 50 μl of blood was collected into EDTA-coated tubes immediately after the glucose measurement, and plasma was stored at −20°C for the later analysis by Ultrasensitive Mouse Insulin ELISA from Mercodia (Uppsala, Sweden). The HOMA index was calculated using the formula [blood glucose (mg/dl) * plasma insulin (μU/ml)/405].

For the glucose tolerance test, blood glucose was measured in the morning in mice fasted for 8 hours. Immediately thereafter, glucose solution (33% w/v; 1.5 g/kg body weight) was injected intraperitoneally. The excursion of blood glucose was documented 20, 40, 60, 90, and 120 min after glucose administration.

### Terminal sample collection

For the terminal collection of plasma and tissue samples, ~4-hour fasted mice were anesthetized with inhalative isoflurane. After confirming deep anesthesia by the absence of the toe pinch reflex, ~500 to 800 μl of blood was collected by heart puncture into syringes prepared with EDTA. Thereafter, tissue specimens were rapidly collected and immediately frozen in liquid nitrogen. Plasma and tissue samples were stored at −80°C until used for analysis.

### Surgical implantation of venous infusion catheters

Surgical implantation of venous catheters was under anesthesia with intraperitoneal injection of saline containing medetomidine hydrochloride (30 mg/liter), midazolam (150 mg/liter), fentanyl (3 mg/liter), and ketamine hydrochloride (4 g/liter) (injection volume: 10 μl/g). Depth of anesthesia was checked repeatedly by toe pinch reflex with additional dosing as required. The right jugular vein was exposed and pricked, and a tapered silicon tube was inserted and pushed 10 mm into the vein (inside diameter: 0.30 mm, outer diameter: 0.64 mm; Dow Corning/Silastic, Midland, MI, USA). The catheter was fixed in this position and subcutaneously guided to the back of the mouse, where it was connected to a subcutaneous port. The catheter was filled with heparin solution (500 U/ml) and remained closed with a cap, until an experiment was performed. After suturing, the mouse received subcutaneous injections of flumazenil (10 mg/liter) plus atipamezole hydrochloride (100 mg/liter) in saline (to antagonize anesthesia), buprenorphine (6 mg/liter) in saline (for analgesia), and 1.5% (w/v) glucose solution (10 μl/g each). During the initial 2 days after surgery, drinking water was supplemented with piritramide (60 mg/liter) and 0.4% (w/v) glucose, and hypoxia-treated individuals remained at normal air. Mice provided with venous catheters were always single housed, and an experiment was not performed earlier than 5 days after surgery.

### Euglycemic-hyperinsulinemic clamp

In the morning, food was withdrawn from mice carrying a venous catheter. Approximately 1 hour later, they were briefly (<1 min) anesthetized with inhalative isoflurane for attaching the infusion tube to the catheter port. Another 30 min later (= time point −120 min), the infusion tube was connected to the infusion line and an infusion of d-[3-^3^H]-glucose (catalog no. NET331C001MC; PerkinElmer, Waltham, MA, USA) started (100 μCi/ml in saline; 20 μl/min during the initial 5 min, 1 μl/min thereafter). Heparinized blood was collected from untreated donor mice, which were on the same diet as the mice used in the experiment (blood sampling procedure as described above in the section “Terminal sample collection”). The solution for erythrocyte infusion was prepared by two rounds of centrifugation (16,000*g*; 1 min) with repeated replacement of the supernatant with an equal volume of heparinized saline (10 U/ml).

At time points −20, −10, and 0 min, blood glucose was measured and 50 μl of blood was collected from the tip of the tail for the determination of insulin and the specific activity of glucose in plasma. At 0 min, the infusion rate of d-[3-^3^H]-glucose was doubled to 2 μl/min, and infusions were started of donor erythrocytes (4 μl/min) and human insulin (Sigma-Aldrich; 0.66 U/ml in saline containing 3% mouse plasma from donor mice; 6 μl/min for 1 min, 0.4 μl/min ≈ 6 mU kg^−1^ min^−1^ thereafter). Donor erythrocytes were infused at the same rate in hypoxia-treated and control mice to compensate for blood loss during the experiment. In that way, not more than 13% of red blood cells were replaced in the course of the clamp, excluding relevant influence on the hematocrit difference between the mouse groups. After starting the insulin infusion, blood glucose was measured at 10-min intervals and a 33% (w/v) glucose solution was infused at a variable rate to maintain blood glucose as close as possible to 7.5 mM. At 90, 100, 110, and 120 min, additional tail blood was sampled for the measurement of insulin and glucose specific activity in plasma. Plasma was stored at −20°C until analyzed.

The experiment then continued with a bolus injection of 40 μl of saline with 10 μCi of 2-[1-^14^C]-deoxy-d-glucose via the catheter (catalog no. NEC495A250UC; PerkinElmer). Two minutes later, a blood sample was secured for measuring 2-[1-^14^C]-deoxy-d-glucose in plasma. After proceeding with insulin and variable glucose infusion for another 30 min, the mouse was euthanized by intravenous injection of a lethal dose of ketamine hydrochloride at time point 150 min. Tissue specimens were rapidly prepared, frozen in liquid nitrogen, and stored at −80°C.

For the determination of d-[3-^3^H] glucose in plasma, samples were deproteinated with zinc sulfate and barium hydroxide. After centrifugation (13,000*g*; 10 min), the supernatant was dried overnight under a stream of nitrogen and glucose was resuspended in water for measuring ^3^H in the scintillation counter. Under baseline conditions, endogenous glucose production equals the glucose turnover rate, which was determined from the ratio of the d-[3-^3^H] glucose tracer infusion rate and the specific activity of plasma glucose. During the clamp period, endogenous glucose production was calculated by subtracting the exogenous glucose infusion rate from the glucose turnover rate, which, in a steady state, equals the rate of glucose uptake.

Plasma ^14^C-2-deoxy-d-glucose 2 min after injection was counted in a manner analogous to the procedure as described above for determination of d-[3-^3^H] glucose. For the measurement of ^14^C-2-deoxy-d-glucose and ^14^C-2-deoxy-d-glucose-6-phosphate in tissues, the specimens were lysed in 1 M NaOH (80°C for 2 hours). After neutralization with 1 M HCl and centrifugation (13,000*g*; 10 min), a small aliquot was used to determine the protein concentration. From the remaining sample, an aliquot was deproteinated with zinc sulfate and barium hydroxide, whereas another aliquot was admixed to a fivefold larger volume of 0.3 M HClO_4_. After centrifugation (13,000*g*; 10 min), ^14^C in the supernatant was counted in the scintillation counter. The results were normalized to protein and to the ^14^C-2-deoxy-d-glucose activity found in plasma 2 min after injection.

### Infusion of erythrocytes or alginate

For erythrocyte infusion, mice carrying a venous catheter were fasted and weighed, and their blood glucose was measured in the morning. They were allocated to erythrocyte or vehicle infusion so to obtain groups matched for weight and glycemia. Thereafter, mice were attached to the infusion tubes and erythrocytes from donor mice were collected as described above in the section “Euglycemic-hyperinsulinemic clamp.” Not earlier than 45 min after connecting the infusion tube, erythrocytes in heparinized saline (10 U/ml) or the vehicle alone were infused at a rate of 12.5 μl/min for 80 min. Blood glucose was measured 30, 60, and 80 min after starting the infusion. The infusion tube was then removed, and under continued fasting, mice were allowed to rest for 2 hours, before a glucose tolerance test was performed (as described above in the section “Glycemia, insulinemia, HOMA, and glucose tolerance test”). Immediately after the test, their hematocrit was determined and mice were provided with food. On the next morning, blood glucose was measured in the fed state and again after 4 hours of fasting. Then, mice were euthanized, and blood was sampled by heart puncture for viscosity measurements at 37°C and at shear rates ranging from 1 to 1000 s^−1^ (Physica MCR301 rheometer; Anton Paar, Austria).

Another experiment followed the same protocol but with infusion of sodium alginate (sodium alginate from Sigma-Aldrich; 15 g/liter in saline) versus vehicle (saline) and with euthanasia and terminal blood sampling immediately after the infusion. In this case, viscosity was determined at 20°C and at a single shear rate of 276 s^−1^ (HAAKE MARS iQ Rheometer, Thermo Fisher Scientific, Waltham, MA, USA).

### Surgical implantation of intracerebroventricular infusion catheters

Under anesthesia as described above in the section “Surgical implantation of venous infusion catheters,” cannulas were surgically implanted into the third ventricle of the brain. To this end, mice were placed in a stereotactic frame (Lab Standard stereotaxic frame; Stoelting, Wood Dale, IL, USA) and their head was shaved. The cranial surface was exposed and cleaned from adhesive tissue through an ~0.6-cm-long midline sagittal incision. After drilling a hole at the appropriate location, a 28-gauge osmotic pump connector cannula for intracerebroventricular infusion (PlasticsOne, Roanoke, VA, USA) was positioned targeting the third ventricle (coordinates: 1.6 mm posterior from bregma on the sagittal suture and −5.5 mm below the cortical surface; source: http://mbl.org/mbl_main/atlas.html). The cannula was fixed to the skull with luting cement (FujiCEMTM 2 SL Automix, Radiopaque Reinforced Glass Ionomer Luting Cement; GC International AG, Luzern, Switzerland), and the intracerebroventricular cannula was connected with vinyl catheter tubing to an osmotic minipump (ALZET, Cupertino, CA, USA; Alzet Model 2006, infusion rate = 0.15 μl/hour), which was filled with rhEPO diluted in artificial cerebrospinal fluid (Tocris, Bristol, UK) so to obtain a continuous infusion of 5 U/kg rhEPO per day. The pump was placed in a subcutaneous pouch in the neck region, which was then sutured. Control mice were analogously implanted with pumps containing artificial cerebrospinal fluid only.

### Soleus muscle incubation ex vivo

Eight-hour fasted mice were briefly anesthetized with inhalative isoflurane and killed by cervical dislocation. Soleus muscles from both legs were immediately prepared, weighed, tied on stainless steel clips, and placed one each into flasks provided with 3 ml of Cell Culture Medium 199 (catalog no. M-4530, Sigma-Aldrich; pH 7.35, 5.5 mM glucose), which was supplemented with 0.3% (w/v) bovine serum albumin, 5 mM Hepes, 300 μM palmitate, and 0.25% (v/v) ethanol (used to dissolve palmitate). Flasks were placed in a shaking water bath (37°C; 130 cycles/min) under an atmosphere of 95% O_2_:5% CO_2_. After 30 min, muscle strips were transferred for 90 min to another set of flasks containing fresh medium with 1 or 100 nM human insulin (Sigma-Aldrich; referred to as basal and insulin-stimulated conditions), as well as with trace amounts of d-[U-^14^C]glucose (catalog no. NEC042V250UC; PerkinElmer), or, for the contralateral muscle from the same mouse, with [U-^14^C]palmitate (catalog no. NEC534050UC; PerkinElmer). Rates of CO_2_ production from glucose or palmitate were calculated from ^14^CO_2_ trapped during the last hour with a solution containing methanol and phenethylamine (1:1), which was analyzed for ^14^C content in the scintillation counter.

### Tissue triglyceride extraction

Triglycerides were extracted from the liver and muscle with a modified chloroform-methanol Folch extraction (*72*). Approximately 100 mg of frozen tissue was homogenized in 1 ml of a mixture of chloroform:methanol (3:1). After adding another 2 ml of the mixture, samples were transferred to glass tubes and incubated for 4 hours at room temperature on a horizontal roller. Saline (1.5 ml) was added, and the samples were vortexed for 10 s and centrifuged at room temperature (940*g*; 10 min). The lower organic phase was transferred to another glass tube and evaporated under a stream of nitrogen. After saponification in 200 μl of 65% ethanol containing 3 M potassium hydroxide (60 min at 70°C), the triglyceride concentration was measured with a Serum Triglyceride Determination Kit (Sigma-Aldrich).

### RNA sequencing and bioinformatics analysis

RNA was extracted from gastrocnemius muscle, liver, and epidydimal adipose tissue derived from hypoxia-exposed mice and their weight-matched controls using TRIzol (Invitrogen, Waltham, MA, USA; *n* = 4 per group). The samples were subjected to transcriptome analysis at the Core Facility RNA Genomics of the Medical University of Vienna (Vienna, Austria). RNA quality was checked on a Bioanalyzer 2100 (Agilent Technologies, Santa Clara, CA, USA). Libraries were prepared using the NEBNext Ultra Directional RNA Library Prep Kit for Illumina (New England Biolabs, Ipswich, MA, USA) according to the manufacturer’s protocols. Libraries were quality control checked on the Bioanalyzer 2100 using a High Sensitivity DNA Kit for correct insert size and quantified using Qubit dsDNA HS Assay (Thermo Fisher Scientific, Waltham, MA, USA). Pooled libraries were sequenced using a NextSeq550 instrument (Illumina, San Diego, CA, USA) in 1x 75–base pair single-end sequencing mode. Data were analyzed on the Illumina BaseSpace platform using the tophat version 2.1.0 and bowtie2 version 2.2.6 for alignment (*73*, *74*) and DESeq2 version 1.6.3 (*75*). Biological insights concerning the differentially expressed genes were explored via GSEA. The analysis was performed with the GSEA software (*76*) (version 2.1.0) using the c2 (version 5) gene set database. Gene sets were considered significantly enriched with a nominal *P* value of <0.05.

### PCR-DNA

For the estimation of mitochondrial density, we used frozen tissue aliquots of the gastrocnemius muscle and liver. Samples were homogenized in DNA isolation buffer [110 mM tris-HCl (pH 8.5), 0.5 M EDTA, 10% SDS, and proteinase K (200 μg/m) from Invitrogen] using an Elvehjem potter with polytetrafluoroethylene pestle, before incubation for at least 3 hours at 37°C. Lysates were then incubated for 10 min at 99°C for inactivation of proteinase K, and DNA was precipitated by incubating the lysates with equal volumes of isopropanol for 15 min under constant shaking at room temperature. To pellet the precipitate, samples were centrifuged for 10 min at 4°C and 15,000*g*. After discarding the supernatant and air-drying, pellets were redissolved in an appropriate volume of TE [10 mmol tris-HCl and 0.1 mM EDTA (pH 7.5)]. To estimate mitochondrial density, the ratio of mitochondrial DNA (mt-Nd2; AGGGATCCCACTGCACATAG = forward primer; CTCCTCATGCCCCTATGAAA = reverse primer) to nuclear DNA (g-Ndufv1; CTTCCCCACTGGCCTCAAG = forward primer; CCAAAACCCAGTGATCCAGC = reverse primer) was analyzed via real-time quantitative PCR (RT-qPCR) using the iTaq Universal SYBR Green Supermix (Bio-Rad Laboratories, Hercules, CA, USA) with the following settings: 2 min at 50°C, 10 min at 94°C, 40x (15 s at 95°C, 15 s at 58°C, and 30 s at 72°C), and 5 s at 60°C, 95°C with the C1000 Thermal Cycler CFX96TM Real Time System (Bio-Rad).

### PCR-RNA

RT-qPCR assays for the measurement of the expression of selected genes involved in the regulation of mitochondrial biogenesis were performed in 384-well reaction plates on a Real-Time PCR System with TaqMan probes (Thermo Fisher Scientific) for *Nrf1* (Assay ID Mm01135606_m1), *Ppargc1a* (Assay ID Mm01208835_m1), *Ppargc1b* (Assay ID Mm00504720_m1), *Co4i1* (Assay ID Mm01250094_m1), *Tfam* (Assay ID Mm00447487_m1), and *Phb* (Assay ID Mm01627033_g1). *Tpb* (liver; Assay ID Mm01277042_m1) and *Actb* (muscle; Assay ID Mm02619580_g1) were used as housekeeping genes. New TaqMan probes were diluted to 1x probes with 250 μl of nuclease free water. A solution of 2.6 μl of nuclease-free water, 0.4 μl of TaqMan probe, and 4 μl of 2x GoTaq Probe qPCR Master Mix (17 μl of CXR reference dye/1.5 ml of Master Mix; Promega, Madfison, WI, USA) was mixed, and 7 μl of the mix pipetted into each well where 1 μl of cDNA was added directly. The plate was centrifuged for 1 min at 115*g*. The PCR program was as follows: 1 cycle of GoTaq activation (2 min at 50°C and 10 min at 95°C) continued by 40 cycles of alternating temperatures (15 s at 95°C and 1 min at 60°C). Genes of interest were pipetted in duplicate and the housekeeping genes in quadruplicate. The melting curves were checked visually. A pool of all samples in equivalent ratios was loaded on each plate for intraassay comparison. The cycle threshold (Ct value) was analyzed with the delta-delta-Ct method [2^(−ddCt)].

### Analysis of blood and plasma samples

Blood glucose was measured with a handheld glucose meter specifically adapted for use in laboratory rodents (StatStrip Xpress; Nova Biomedical, Waltham, MA, USA; adapted for use in rodents by Data Sciences International, St. Paul, MN, USA). At variance to most other handheld strip glucose meter devices, the used StatStrip Xpress has been documented to be insensitive to the hematocrit of the blood sample in a range of at least 25 to 70% (*18*–*22*). To further assure hematocrit insensitivity of the used glucose meter, the decrease in blood glucose found in hypoxia-treated mice was carefully confirmed by measurements of glucose concentration in their blood and plasma with a cobas c 111 analyzer (Roche Diagnostics, Basel, Switzerland), in blood with an ABL800 FLEX Analyzer (Drott Medizintechnik, Wiener Neudorf, Austria), and in plasma with the Glucose liquiUV mono kit from HUMAN Diagnostics (Ahrensburg, Germany). In the blood samples examined, results from all devices consistently showed a hypoxia-induced decrease in blood and plasma glucose by ~20%, whereas the values obtained with the handheld glucose meter OneTouch Ultra (LifeScan Inc., Milpitas, CA, USA) deviated from what was found with the other devices (decrease by ~30%), which was in accordance with its previously reported hematocrit dependence (*77*).

Commercially available assay kits and a multimode microplate reader were used for the measurement in plasma of insulin (Mercodia, Uppsala, Sweden), leptin (Phoenix Pharmaceuticals Burlingame, CA, USA), adiponectin (Merck Millipore, Burlington, MA, USA), EPO (Abcam, Cambridge, UK), FGF-21 (R&D Systems, Minneapolis, MN, USA), triglycerides (Sigma-Aldrich), and free fatty acids and ketone bodies (both FUJIFILM Wako Chemicals Europe GmbH, Neuss, Germany).

A stationary blood gas analyzer was used for the determination of hematocrit, blood oxygen saturation, and blood lactate (ABL800 FLEX Blood Gas Analyzer). The used device does not measure the hematocrit directly but estimates the value from the hemoglobin concentration. To protect mice from sampling of large blood volumes, most measurements of hematocrit and blood oxygen saturation were on pooled blood samples from two arbitrarily selected individuals belonging to the same experimental group.

### Erythrocytes in vitro

Blood was sampled by terminal heart puncture from male obese mice (~8 months old and after 6 months on HFD), and aliquots of 200 μl were cooled on ice and centrifuged at 4°C.

In the first protocol, plasma was removed after centrifugation and the erythrocytes were resuspended in 120 μl of Dulbecco’s PBS (DPBS; no calcium, no magnesium; Gibco, Grand Island, NY, USA) supplemented with 0.55 or 16.5 mM glucose. One hour of incubation under gentle shaking at 37°C was followed by another round of centrifugation and by resuspension of erythrocytes in 120 μl of glucose-free DPBS. Aliquots from the same blood sample were subjected to centrifugation and collection of supernatant immediately after resuspension or after 20 min under gentle shaking at 37°C. The difference (Δ) in glucose and lactate content of the medium before versus after the 20 min of incubation was determined (= net release of glucose and lactate from the erythrocytes).

In the second protocol, obese mice were treated for 2 months with rhEPO (three doses of 300 U/kg per week) or the vehicle (as in the experiments shown in Fig. 2). Erythrocytes were collected from 200 μl-aliquots of their blood. After centrifugation, erythrocytes were resuspended in 120 μl of DPBS supplemented with 1.1 or 22 mM glucose. Again, one aliquot was centrifuged immediately and the supernatant collected, whereas a corresponding aliquot was centrifuged after 20 min of incubation under gentle shaking at 37°C and the supernatant collected. The difference (Δ) in glucose and lactate content of the supernatants before versus after the 20 min of incubation was determined.

Glucose and lactate in the collected supernatants were determined with colorimetric assay kits (Glucose liquiUV mono from Human, Wiesbaden, Germany; and l-Lactate assay kit from Abbott, Chicago, IL, USA). Molar amounts of lactate and glucose in the samples were calculated under the assumptions that the concentration differences between the erythrocyte fraction and the incubation buffer were similar to those between human erythrocyte fraction and plasma (*78*, *79*), as well as that this relation was not affected by the experimental conditions.

### Human data

Data were obtained from examinations performed in the course of compulsory military conscription, which is obligatory for all 18-year-old male Austrians except those unable to participate because suffering from severe mental or physical disorders (thus including >95% of Austrian male adolescents). Acquisition of data within the observational period (2004 to 2019) was performed by the Federal Ministry of Defense with data collection and blood sampling done by well-instructed military service members and along highly standardized procedures. Datasets lacking values for BMI, fasted blood glucose, hematocrit, and/or smoking status, as well as such from subjects with a diagnosis of diabetes mellitus were excluded. To circumvent as far as possible the influence of other specific pathologies and flaws in laboratory analysis, datasets outside the healthy range for fasted blood glucose (3.85 to 6.05 mM) or hematocrit (41 to 52%) were also excluded. The remaining population still contained the bulk of the data (*n* = 497,434), which was retrospectively analyzed for comparison of nonsmokers (*n* = 302,829) to smokers (*n* = 194,605).

### Statistical analysis

Results are reported as means ± SEM. In the context of an exploratory data analysis, two-tailed *t* tests (paired or unpaired, as appropriate) and Pearson’s correlation were applied. Occasionally mentioned *P* values for outliers are by Grubb’s outlier test. *P* < 0.05 was considered significant.

### Study approval

Experiments and protocols from mouse experiments were critically reviewed by an internal animal ethics committee at the Medical University of Vienna and were approved by an expert committee authorized by the Austrian Federal Ministry of Education, Science and Research. Legal approval was granted by the Ministry (approval nos. BMWFW-66.009/0296-WF/II/3b/2012, BMWFW-66.009/0089-WF/II/3b/2014, BMWFW-66.009/0275-WF/V/3b/2017, and BMWFW-66.009/0379-V/3b/2019).

Procedures for the study of human data were approved by both a local ethics committee and the Austrian Agency for Health and Food Safety (approval no. 2261/2019).
