# Supplementary material for: A direct effect of the hematocrit on blood glucose: Evidence from hypoxia- and erythropoietin-treated mice
Source: Sci Adv. 2025 Apr 16;11(16):eadt7366. doi: 10.1126/sciadv.adt7366 (PMC12002128; doi:10.1126/sciadv.adt7366)
Supplement: Supplementary file 1 — Figs. S1 to S9 [file sciadv.adt7366_sm.pdf]

Science Advances
AAAS

Supplementary Materials for

A direct effect of the hematocrit on blood glucose: Evidence from hypoxia- and erythropoietin-treated mice

Thomas Scherer *et al.*

Corresponding author: Clemens Fürnsinn, clemens.fuernsinn@meduniwien.ac.at

Sci. Adv. **11**, eadt7366 (2025)
DOI: 10.1126/sciadv.adt7366

This PDF file includes:

Figs. S1 to S9

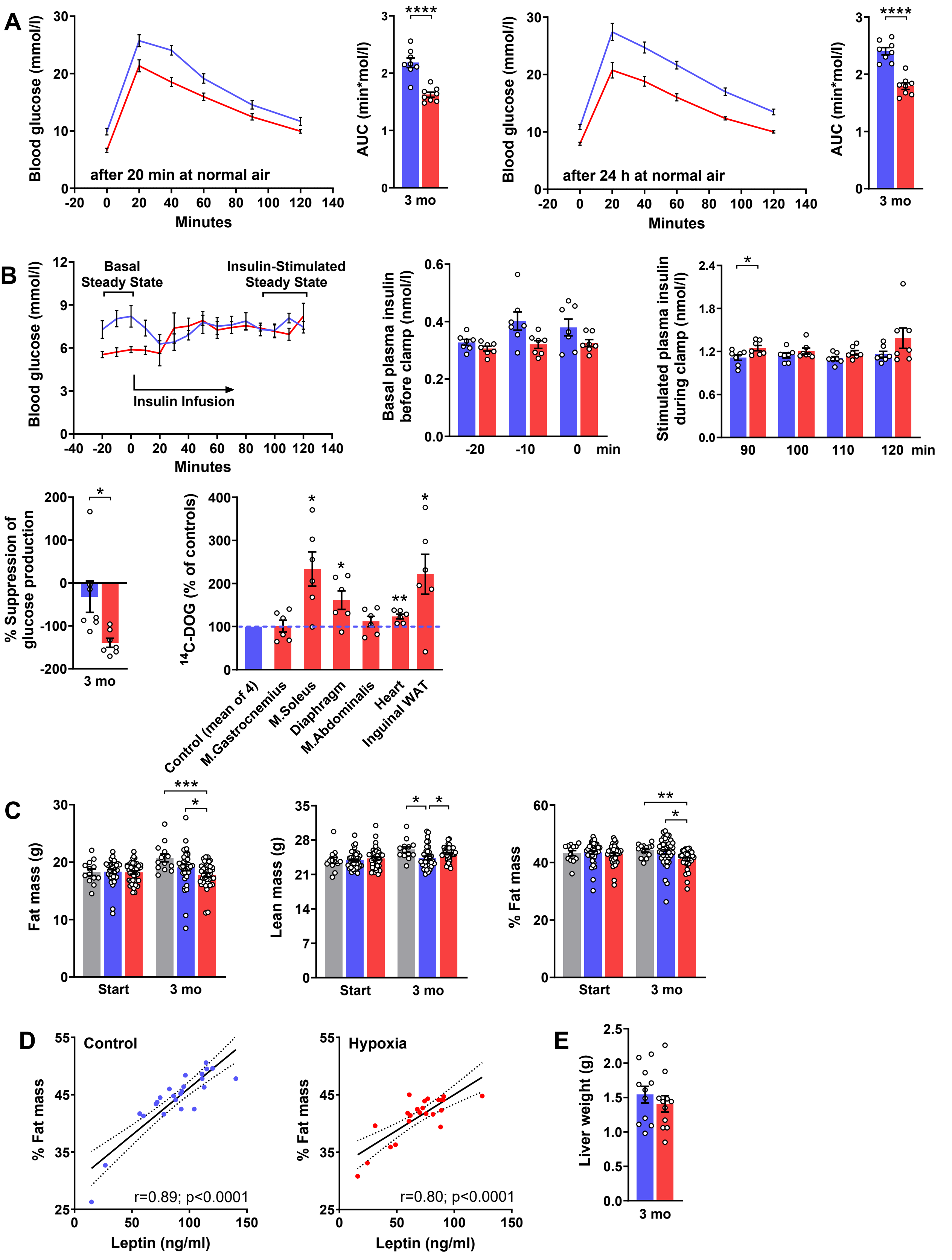

Supplemental Figure S1: Metabolic response to life under hypoxia - supplemental results.
Metabolic characteristics of male obese mice, which after three months on high fat diet were exposed to hypoxia for another three months (10% O_2, red). They were compared to control groups at normal air with free access to food (grey), or with restricted access to food (blue) so to maintain a weight curve mimicking that of the hypoxia-exposed mice (results from the same mice as shown in Fig.1). Graphs depict (**A**) glucose excursion during glucose tolerance tests performed 20 min and 24 h after removal from hypoxia with the corresponding AUC (1.5 g/kg i.p.); (**B**) blood glucose, plasma insulin, relative suppression of glucose production, and accumulation of ^{14}C-deoxy-glucose (^{14}C-DOG) in tissues during a euglycemic-hyperinsulinemic clamp test; (**C**) body composition; (**D**) intra-individual association of % fat mass with plasma leptin in control and weight-matched hypoxia-treated mice; (**E**) liver weight.
Mean±SEM; *p<0.05, **p<0.01, ***p<0.001, ****p<0.0001 by Student´s t test.

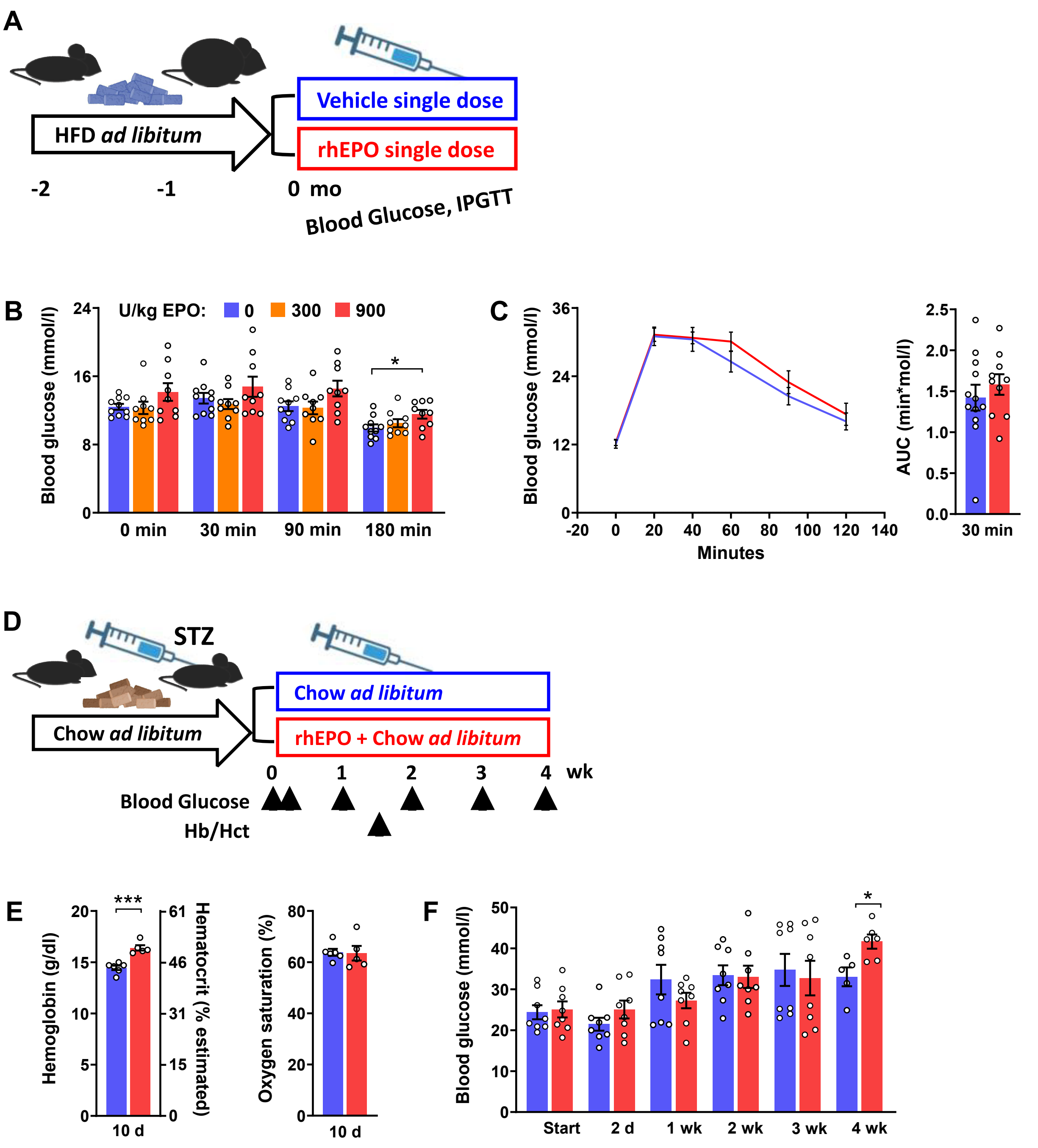

Supplemental Figure S2: Erythropoietin-induced glucose lowering requires repeated treatment and the presence of insulin.

(**A**-**C**) Male obese mice received a single i.p. dose of epoietin theta (rhEPO). Graphs depict (**A**) experimental protocol; (**B**) basal blood glucose 30, 90, and 180 min after injecting 300 U/kg rhEPO (orange), 900 U/kg rhEPO (red), or the vehicle (blue); (**C**) glucose excursion during a glucose tolerance test with corresponding AUC (1.5 g/kg i.p) started 30 min after injecting 500 U/kg rhEPO (red) or vehicle (blue).
(**D**-**F**) Male lean mice with streptozotocin (STZ)-induced insulin deficiency were treated for one month with three i.p. doses per week of 300 U/kg rhEPO (red) or the vehicle (blue). Graphs depict (**D**) experimental protocol; (**E**) hemoglobin/hematocrit and blood oxygen saturation (on day 10 of rHEPO treatment); (**F**) basal blood glucose.
Mean±SEM; *p<0.05 by Student´s t test.

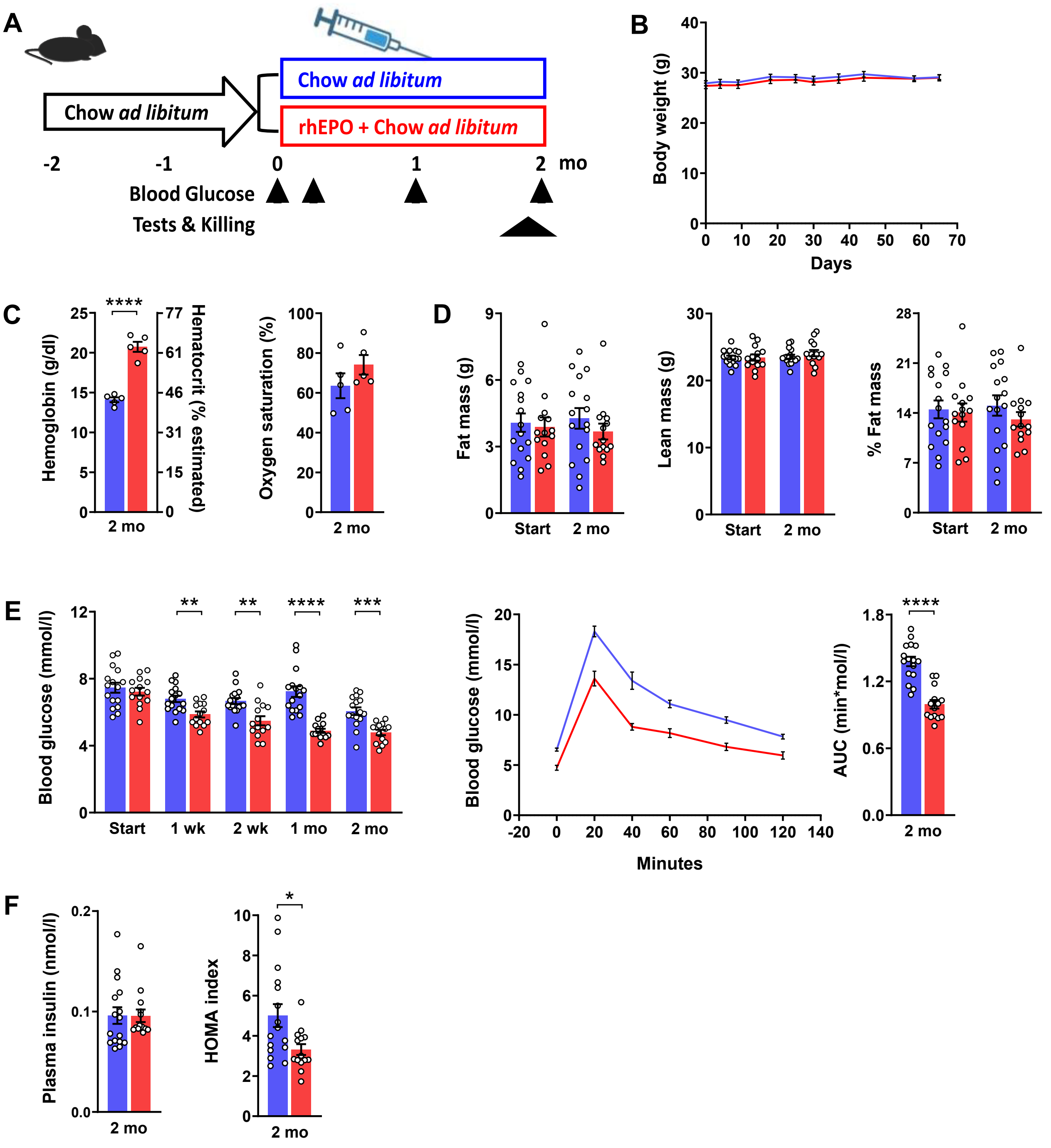

Supplemental Figure S3: Erythropoietin lowers blood glucose in lean mice.
Metabolic characteristics of male lean mice on chow diet, which were treated for two months with three i.p. doses per week of 300 U/kg epoietin theta (rhEPO; red) or the vehicle (blue). Graphs depict (**A**) experimental protocol; (**B**) weight curves; (**C**) hemoglobin/hematocrit and blood oxygen saturation; (**D**) body composition; (**E**) basal blood glucose and glucose excursion during a glucose tolerance test with corresponding AUC (1.5 g/kg i.p); (**F**) plasma insulin and HOMA index.
Mean±SEM; *p<0.05, **p<0.01, ***p<0.001, ****p<0.0001 by Student´s t test.

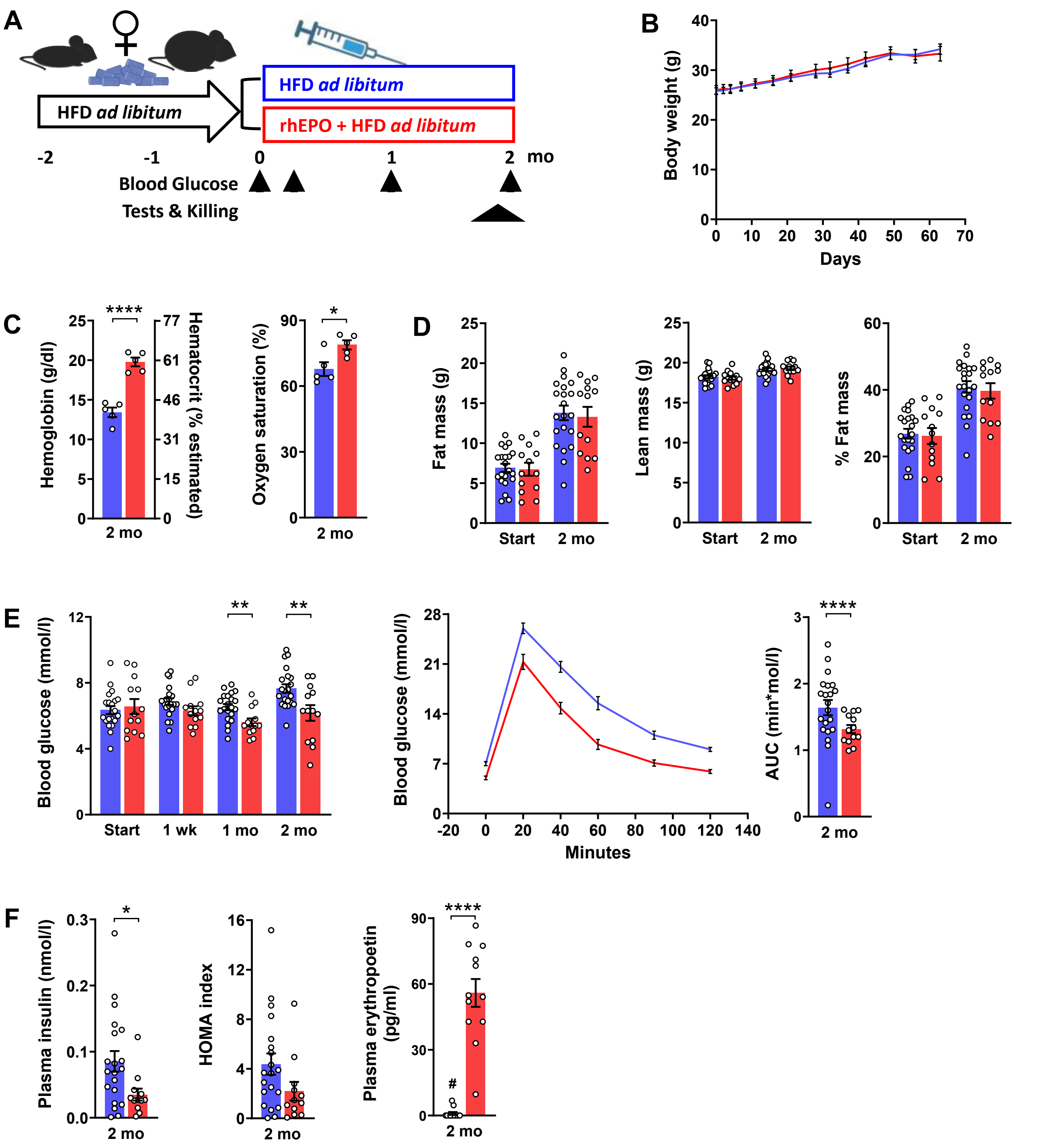

Supplemental Figure S4: Erythropoietin lowers blood glucose in female mice.
Metabolic characteristics of female obese mice, which after two months on high fat diet (HFD) were treated for another two months with three i.p. doses per week of 300 U/kg epoietin theta (rhEPO; red) or the vehicle (blue). Graphs depict (**A**) experimental protocol; (**B**) weight curves; (**C**) hemoglobin/hematocrit and blood oxygen saturation; (**D**) body composition; (**E**) basal blood glucose and glucose excursion during a glucose tolerance test with corresponding AUC (1.5 g/kg i.p); (**F**) plasma insulin, HOMA index, and plasma erythropoietin (# below detection limit in 10 out of 12 control mice).
Mean±SEM; *p<0.05, **p<0.01, ***p<0.001, ****p<0.0001 by Student´s t test.

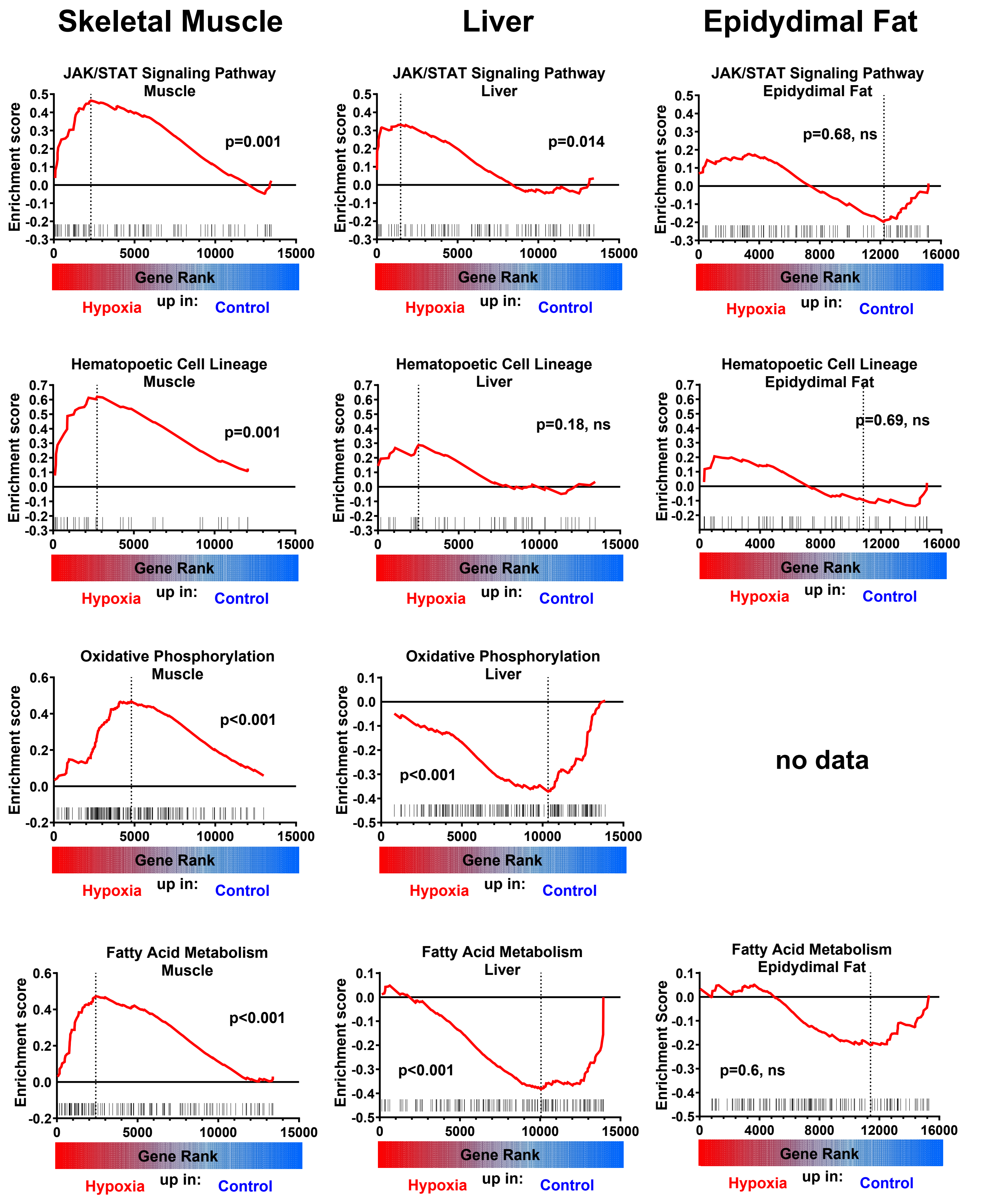

Supplemental Figure S5: Effects of hypoxia on gene expression by gene set enrichment analysis.
Shown are gene enrichment plots for skeletal muscle, liver, and epidydimal fat from male obese mice, which after three months on high fat diet were exposed to hypoxia for another three months (10% O_2, red), as compared to a control group at normal air with restricted access to food so to maintain a weight curve mimicking that of the hypoxia-exposed mice (blue). Gene enrichment plots are shown for gene sets related to the JAK/STAT signaling pathway and to the hematopoietic cell lineage (KEGG pathway analysis), as well as for gene sets related to oxidative phosphorylation and fatty acid metabolism (Hallmark pathway analysis).

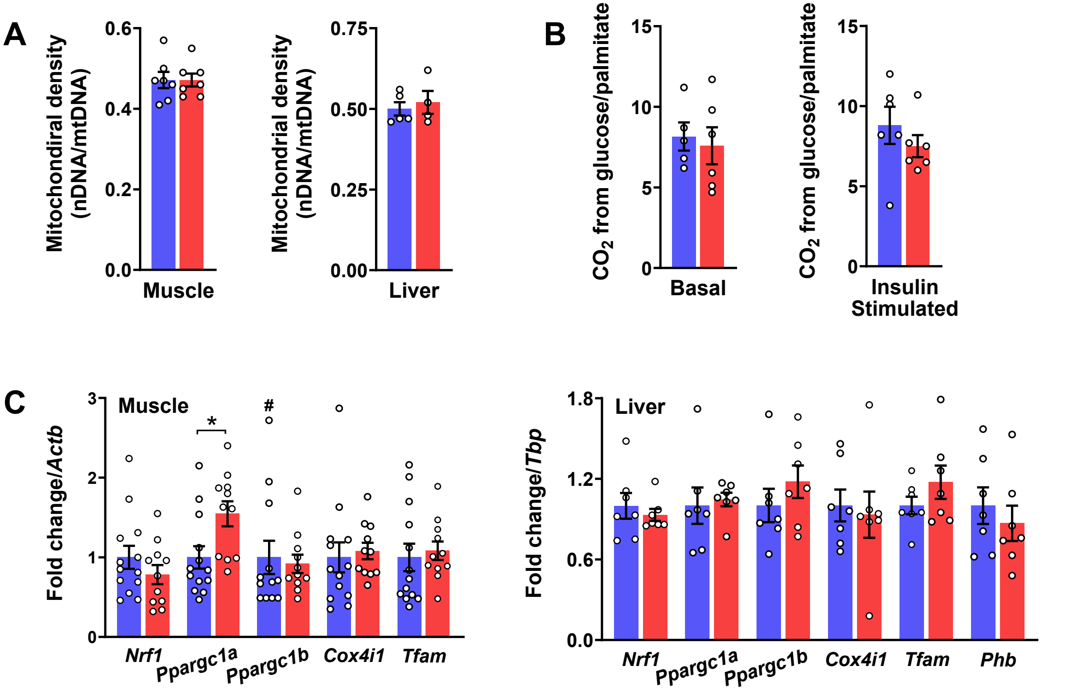

Supplemental Figure S6: Hypoxia-induced glucose lowering does not involve mitochondrial biogenesis.
Parameters indicative of mitochondrial biogenesis in tissue specimens from male obese mice, which after three months on high fat diet were exposed to hypoxia for another three months (10% O_2, red), as compared to controls at normal air with restricted access to food so to maintain a weight curve mimicking that of the hypoxia-exposed mice (blue). Graphs depict (**A**) mitochondrial density in muscle and liver (nuclear/mitochondrial DNA); (**B**) oxidative fuel selection (glucose/palmitate) by skeletal muscle *ex vivo*; (**C**) expression of mitochondrial biogenesis genes in muscle and liver (# one outlier of 4.96 is outside the depicted range; outlier $p<0.0001$).
Mean±SEM; *$p<0.05$ by Student´s t test.

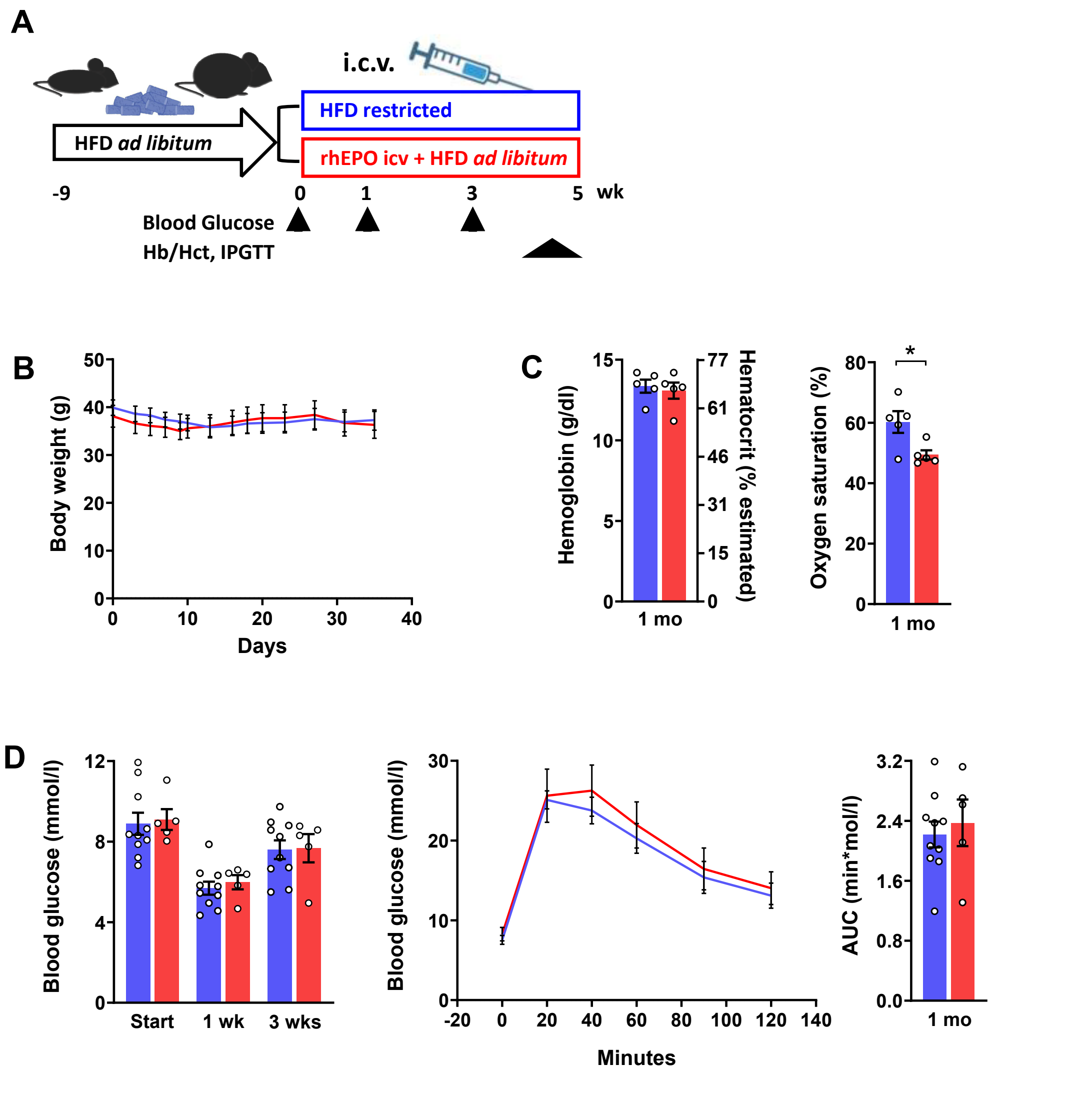

Supplemental Figure S7: No evidence for erythropoietin action on blood glucose via the brain. Metabolic characteristics of male obese mice, which after two months of high fat feeding (HFD) were continuously infused into the 3rd ventricle of the brain (i.c.v.) with 5 U/kg per day of epoietin theta (rhEPO) for one month (red). Controls were infused with the vehicle (blue). Graphs depict (**A**) experimental protocol; (**B**) weight curves; (**C**) hemoglobin/hematocrit and blood oxygen saturation; (**D**) basal blood glucose and glucose excursion during a glucose tolerance test with corresponding AUC (performed on day 30; 1.5 g/kg i.p).
Mean±SEM; *p<0.05 by Student´s t test.

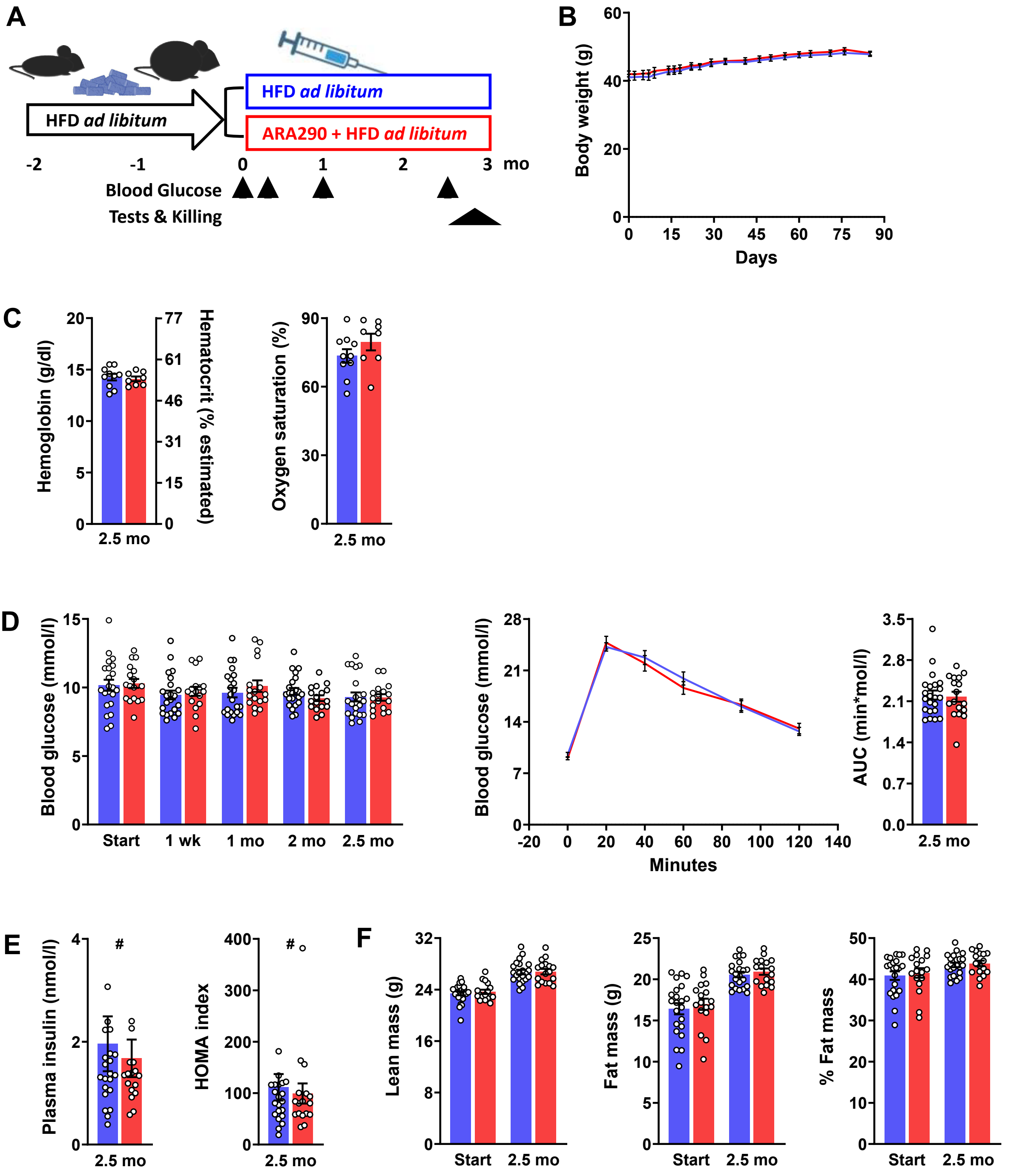

Supplemental Figure S8: ARA290 does not affect blood glucose.

Metabolic characteristics of male obese mice, which after two months on high fat diet (HFD) were for three months treated with three i.p. doses per week of ARA290 (75 µg/kg during initial month, 150 µg/kg thereafter; red), or the vehicle (blue). Graphs depict (**A**) experimental protocol; (**B**) weight curves; (**C**) hemoglobin/hematocrit and blood oxygen saturation; (**D**) basal blood glucose and glucose excursion during a glucose tolerance test with corresponding AUC (1.5 g/kg i.p); (**E**) plasma insulin and HOMA index; (**F**) body composition.

Mean±SEM; no significant differences control *versus* ARA290; # one ARA290-treated outlier is outside the depicted range (plasma insulin 7.21 nmol/l; outlier $p<0.0001$).

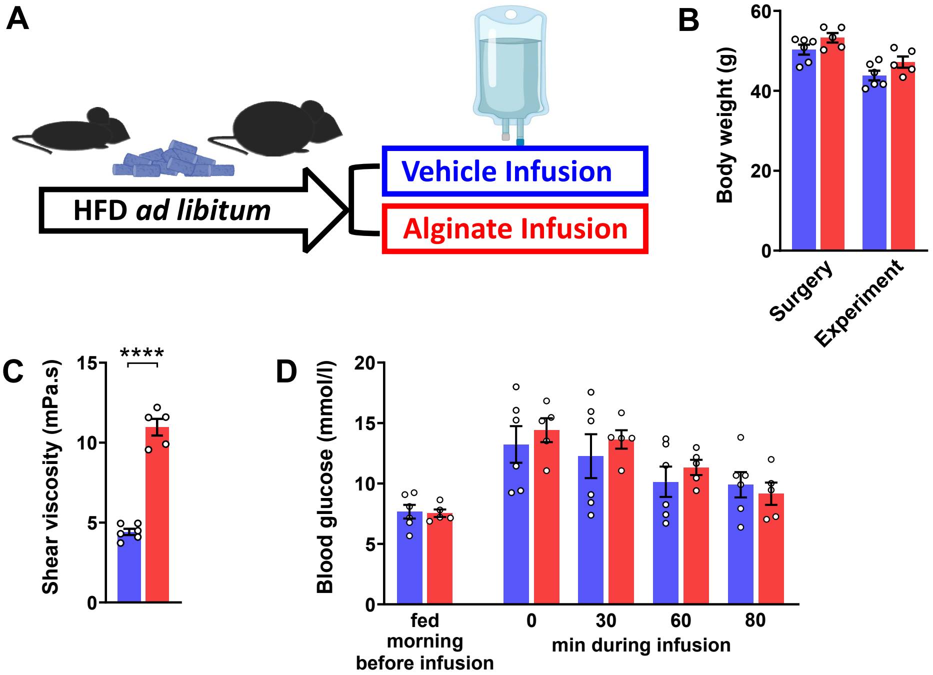

Supplemental Figure S9: Alginate infusion does not affect blood glucose.
Metabolic characteristics of male obese mice, which after three months of high fat feeding (HFD) were infused with alginate (red). Controls were infused with the vehicle (blue). Graphs depict (**A**) experimental protocol; (**B**) body weight at surgery and at experiment; (**C**) shear viscosity at a shear rate of 276 s^{-1} of the blood immediately after infusion; (**D**) blood glucose on the morning before, during and immediately after the 80 min infusion period.
Mean±SEM; ****p<0.0001 by Student´s t test.
